# Disruption of undecaprenyl phosphate recycling suppresses *ampC* beta-lactamase induction in *Pseudomonas aeruginosa*

**DOI:** 10.1371/journal.ppat.1013633

**Published:** 2025-10-21

**Authors:** Karina Klycheva, Joël Gyger, Mélissa Frund, Gabriel Torrens, Felipe Cava, Coralie Fumeaux

**Affiliations:** 1 Institute of Microbiology, Lausanne University Hospital and University of Lausanne, Lausanne, Switzerland; 2 Department of Molecular Biology and Laboratory for Molecular Infection Medicine Sweden, Umeå Centre for Microbial Research, SciLifeLab, Umeå University, Umeå, Sweden; Nanyang Technological University, SINGAPORE

## Abstract

Beta-lactam antibiotics are widely used to treat bacterial infections, but their efficacy is compromised by resistance mechanisms such as the production of beta-lactamases. In *Pseudomonas aeruginosa*, the chromosomally encoded beta-lactamase AmpC is the primary mediator of beta-lactam resistance. *ampC* expression is regulated by the transcription factor AmpR, which responds to intracellular peptidoglycan (PG) fragments. Under normal conditions, AmpR binds the PG precursor (UDP-MurNAc-pentapeptide) and represses *ampC* expression. However, during beta-lactam treatment or in PG recycling-deficient mutants such as *ampD* mutants, PG degradation products (anhydromuropeptides) accumulate and activate AmpR, resulting in elevated *ampC* expression and beta-lactam resistance. We hypothesized that shifting the balance of PG precursors could modulate AmpR activity and suppress beta-lactamase expression, even in derepressed strains. Undecaprenyl phosphate (UndP) is a lipid carrier essential for translocating PG precursors across the bacterial inner membrane. Recent work has identified members of the DedA superfamily as UndP flippases responsible for recycling this lipid carrier. Disruption of UndP recycling leads to cytoplasmic accumulation of UDP-MurNAc-pentapeptide, the known AmpR repressor. Here, we show that deletion of *dedA4*, which encodes a predicted UndP flippase in *P. aeruginosa*, causes PG precursors accumulation and significantly reduces AmpC production and beta-lactam resistance in an *ampD* mutant. These findings highlight the influence of PG precursor dynamics on beta-lactamase regulation and identify DedA4 as a promising therapeutic target. Inhibiting UndP recycling offers a novel strategy to counteract beta-lactam resistance in *P. aeruginosa* and potentially other AmpC-producing pathogens.

## Author summary

Beta-lactam resistance remains a major clinical challenge, often driven by the overexpression of chromosomally encoded beta-lactamases such as AmpC in *Pseudomonas aeruginosa*. Strategies to limit *ampC* activation are urgently needed to preserve the efficacy of these frontline antibiotics. Our study identifies DedA4, a membrane protein involved in undecaprenyl phosphate (UndP) recycling, as a potential target for such intervention. Inactivation of DedA4 disrupts UndP transport, leading to the accumulation of soluble peptidoglycan precursors which act as corepressors of AmpC expression. This disruption in the balance between cell wall synthesis and turnover restores susceptibility to β-lactams, even in strains harboring clinically significant resistance mutations such as those in *ampD* or *dacB*. Targeting UndP flippases like DedA4 could therefore represent a novel adjuvant strategy to combat AmpC-mediated beta-lactam resistance.

## Introduction

*Pseudomonas aeruginosa* is an opportunistic pathogen that poses a significant clinical challenge due to its intrinsic and acquired mechanisms of antibiotic resistance [1,2]. A major contributor to this resistance is the chromosomally encoded AmpC beta-lactamase, which can hydrolyze a broad range of beta-lactam antibiotics, reducing their therapeutic efficacy [3–5]. The expression of the *ampC* gene is tightly regulated and responsive to cell wall stress, particularly during exposure to certain beta-lactams [5]. Under normal conditions, *ampC* is expressed at low basal levels. However, treatment with beta-lactamase-inducing antibiotics, such as cefoxitin and imipenem, strongly upregulates *ampC*, whereas non-inducers like piperacillin and ceftazidime typically do not [3,4]. Non-inducing beta-lactams retain activity against *P. aeruginosa*, but become ineffective when *ampC* is dysregulated [4,6].

Mutations in cell wall-related enzymes, such as the cytoplasmic amidase AmpD or the periplasmic D,D-endopeptidase/carboxypeptidase DacB [7,8], can lead to constitutive overexpression of *ampC*, resulting in high-level resistance, even to non-inducing beta-lactams. These mutants are clinically relevant, having been isolated from patients and associated with therapeutic failure [5–9]. Consequently, there is growing interest in dissecting the molecular pathways that govern *ampC* expression and identifying strategies to counteract its upregulation.

**Fig 1:**
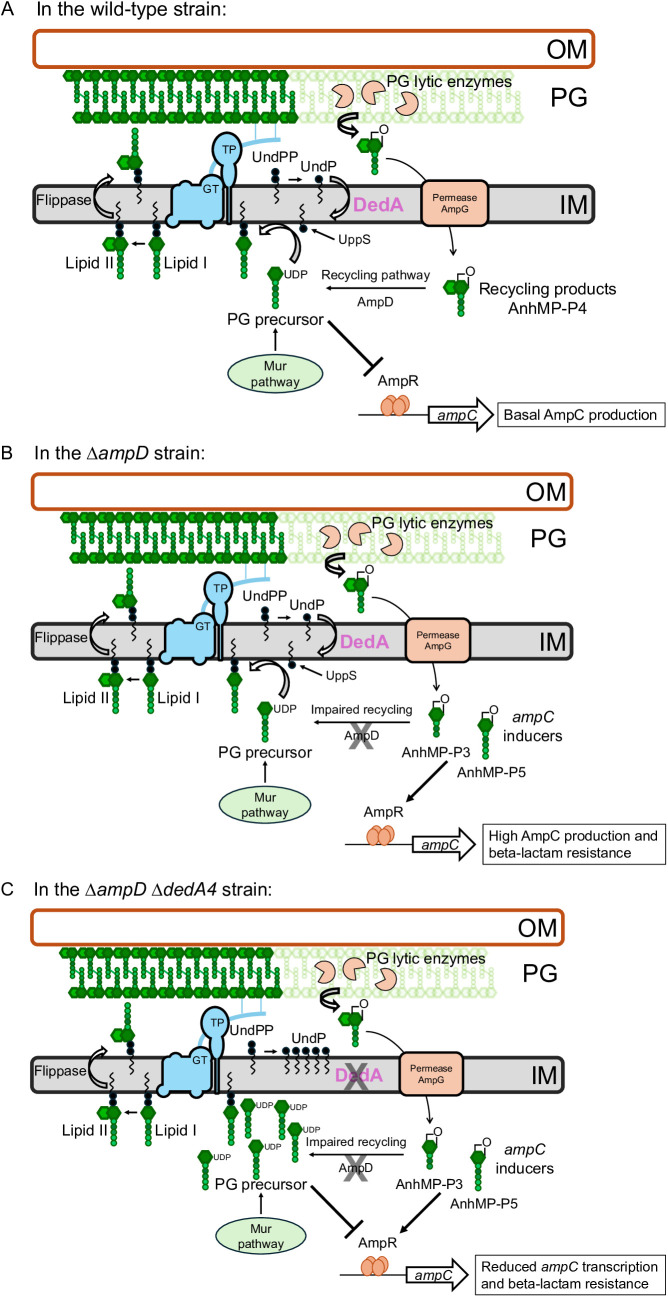
Overview of peptidoglycan (PG) cell wall synthesis, undecaprenyl phosphate (UndP) cycle and *ampC* regulation in *P. aeruginosa* wild-type, ∆*ampD* and ∆ *ampD* ∆ *dedA4 *strains. **(A)** Peptidoglycan precursors are synthesized in the cytoplasm via the Mur pathway and loaded onto the lipid carrier undecaprenyl phosphate (UndP) [16–18]. These UndP-linked precursors are transported across the membrane and incorporated into the expanding PG matrix by glycosyltransferase (GT) and transpeptidase (TP) enzymes [17]. Undecaprenyl pyrophosphate (UndPP), generated in the process, is dephosphorylated and flipped by members of the DedA superfamily of flippases to regenerate cytoplasmic-facing UndP [19,20]. UppS is responsible for *de novo* synthesis of UndP [21]. Mature PG is degraded by PG lytic enzymes to produce anhydro-MurNAc-containing muropeptide (anhMP) turnover products, which are imported into the cytoplasm by the AmpG permease for recycling [5,22]. Tetrapeptide-anhMP products (anhMP-P4) are rapidly converted into anhMP-P3 by the L,D-carboxypeptidase LdcA and then processed by additional recycling enzymes to form new PG precursors. These precursors prevent the activation of *ampC* expression by AmpR, resulting in *ampC* repression in the absence of cell wall damage [11,12]. **(B)** In the *ampD* mutant, PG recycling is impaired, leading to the accumulation of tripeptide-anhMP (anhMP-P3), predominantly, and to a lesser extent pentapeptide-AMP (anhMP-P5) in the cytoplasm. These turnover products are thought to be potent activators of AmpR, resulting in *ampC* induction and beta-lactam resistance [10,11,14,15]. **(C)** Model illustrating the effect of *dedA4* deletion on the balance between PG recycling products and PG precursors, AmpR activity and *ampC* transcription in the ∆*ampD* background (see text for details).

The transcriptional regulator AmpR controls *ampC* expression in response to the balance between specific peptidoglycan (PG) biosynthetic precursors and recycling products [5,9–11]. In wild-type cells, PG turnover fragments are efficiently recycled and AmpR binds cytoplasmic PG precursors (UDP-MurNAc-pentapeptide, UDP-M5), forming a tetrameric complex that represses *ampC* transcription [12,13] (Fig 1A). In contrast, during a beta-lactam treatment or in *ampD* or *dacB* mutants, an abnormal buildup of PG recycling intermediates (anhydromuropeptides, anhMP), switches AmpR into its activator state, leading to constitutive high-level *ampC* expression [5,14,15] (Fig 1B).

We hypothesized that shifting the balance back toward PG precursors, even in derepressed mutants, could suppress *ampC* induction. One potential way to achieve this is by limiting the availability of the lipid carrier undecaprenyl phosphate (UndP), which is essential for PG synthesis [16,18]. In the cytoplasm, PG precursors are synthesized by the MurA-MurF enzymes to form UDP-MurNAc-pentapeptide (UDP-M5). This molecule is transferred to UndP by MraY to form Lipid I, which is then converted into Lipid II by MurG through addition of the second sugar UDP-GlcNAc. Lipid II is flipped across the inner membrane by the flippase MurJ and incorporated into the cell wall by glycosyltransferase and transpeptidase enzymes [17,23]. Once utilized, Lipid II generates undecaprenyl pyrophosphate (UndPP), which undergoes dephosphorylation and recycling back into UndP, ensuring continued PG synthesis [16,18–20,24] (Fig 1).

Recent studies in *Vibrio cholerae* and *Staphylococcus aureus* have shown that deletion of UndP recycling enzymes, members of the DedA superfamily and DUF368 domain-containing proteins, leads to the cytoplasmic accumulation of UDP-M5 [19]. This occurs due to an insufficient pool of UndP, despite ongoing synthesis by the *de novo* pathway via UppS (also named IspU in *Escherichia coli*) [21]. The resulting accumulation of PG precursors may affect cell wall homeostasis and regulatory pathways, including *ampC* induction (Fig 1C).

To test whether UndP recycling affects *ampC* regulation in *P. aeruginosa*, we investigated the impact of deleting its predicted UndP flippase genes. Here, we report the effects of *dedA4* deletion in both wild-type and Δ*ampD* backgrounds. We show that the disruption of UndP recycling leads to UDP-M5 accumulation and modulates AmpR activity. In *ampC*-derepressed strains, this shift in precursor balance suppresses AmpC production and beta-lactam resistance (Fig 1C). Our findings suggest that UndP recycling is a regulatory node in beta-lactam resistance. Inhibiting UndP recycling could represent a novel therapeutic strategy to enhance beta-lactam efficacy against resistant *P. aeruginosa* strains and other AmpC-producing species, like *Citrobacter freundii* and *Enterobacter cloacae* [4].

## Results

### Inactivation of *P. aeruginosa dedA4* reduces *AmpC* production and beta-lactam resistance

As described above (Fig 1), AmpR activity is influenced by cytoplasmic PG precursor levels. Based on this, we hypothesized that disrupting UndP recycling would impact PG precursor levels and reduce *ampC* induction. Unlike some other organisms, *P. aeruginosa* lacks DUF368 homologues but encodes five DedA family members [25]. In *Bacillus subtilis*, the *uptA ykoX* double mutant lacks known UndP flippases, providing a system to test candidate genes for this function [20]. Among the *P. aeruginosa* DedA proteins, DedA4 (PA4029) was previously shown to complement this *Bacillus* mutant, suggesting that it acts as a bona fide UndP flippase [20]. Therefore, we deleted *dedA4* (*PA4029*) in wild-type and ∆*ampD P. aeruginosa* strains and assessed the effects on AmpC levels and beta-lactam resistance.

**Fig 2:**
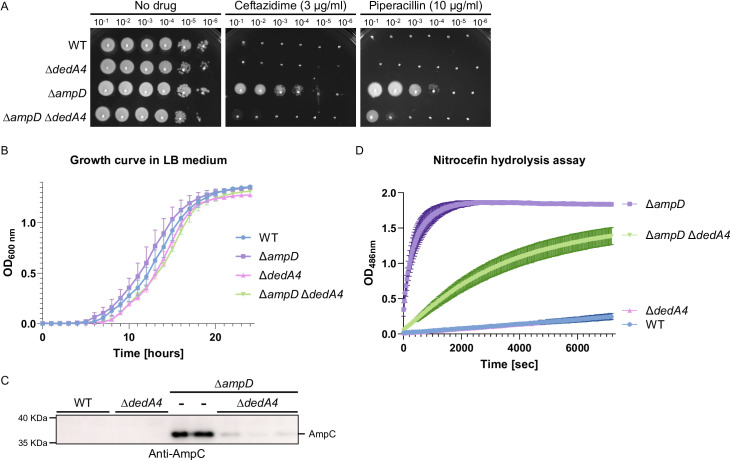
DedA4 is required for AmpC production and beta-lactam resistance in cells defective for AmpD. **(A)** Cultures of strains PAO1 [WT], CF1842 [∆*dedA4*], CF5 [∆*ampD*] and CF1844 [∆*ampD* ∆*dedA4*] were serially diluted and 5 µl of each dilution was spotted onto LB agar supplemented with either ceftazidime (3 µg/ml) or piperacillin (10 µg/ml), as indicated. Plates were incubated overnight at 30^o^C and photographed. **(B)** Growth curve in LB medium recorded for 24h using the strains from panel (A). **(C)** Immunoblot for AmpC protein using the strains from panel (A), including biological replicates. **(D)** Nitrocefin hydrolysis assays using cell lysates of the strains from panels (A). Data represent the mean of three independent assays with two to four biological replicates; error bars indicate standard error.

To monitor the effect of DedA4 inactivation on *ampC* induction, we tested the beta-lactam resistance of mutant strains. Consistent with prior results, the ∆*ampD* strain was highly resistant to the antipseudomonal beta-lactams, ceftazidime and piperacillin (Fig 2A). Deletion of *dedA4* in the ∆*ampD* strain significantly reduced beta-lactam resistance (Fig 2A), while it did not affect viability in the absence of the antibiotic. To assess potential fitness differences, growth curves were measured over 24 hours in LB medium. Both the *dedA4* single mutant and the ∆*ampD* ∆*dedA4* double mutant displayed a slightly extended lag phase (approximately one hour longer than wild type), but the subsequent exponential growth rate and final optical density were comparable to wild type and ∆*ampD* strains (Fig 2B). To quantify the change in resistance, we measured the minimum inhibitory concentrations (MICs) of the relevant strains. We confirmed that the double mutant ∆*ampD* ∆*dedA4* was 4 times more sensitive to ceftazidime and piperacillin than the parental ∆*ampD* strain, with MICs of 20 µg/ml (∆*ampD*) and 5 µg/ml (∆*ampD* ∆*dedA4*) for piperacillin, and 5 µg/ml (∆*ampD*) and 1.25 µg/ml (∆*ampD* ∆*dedA4*) for ceftazidime (Table 1). As controls, we measured MICs for the non-beta-lactam antibiotics A22, tobramycin and gentamicin, which showed no significant changes across strains (Table 1). These results suggest that deletion of *dedA4* impacts specifically resistance to beta-lactam antibiotics but not cell envelope integrity.

**Table 1:** Minimal inhibitory concentration (MIC) of selected antibiotics.

Antibiotics	∆*ampD*	∆*ampD* ∆*dedA4*
Piperacillin	20	5
Ceftazidime	5	1.25
A22	3	3
Tobramycin	2	2
Gentamicin	4	4

**Fig 3:**
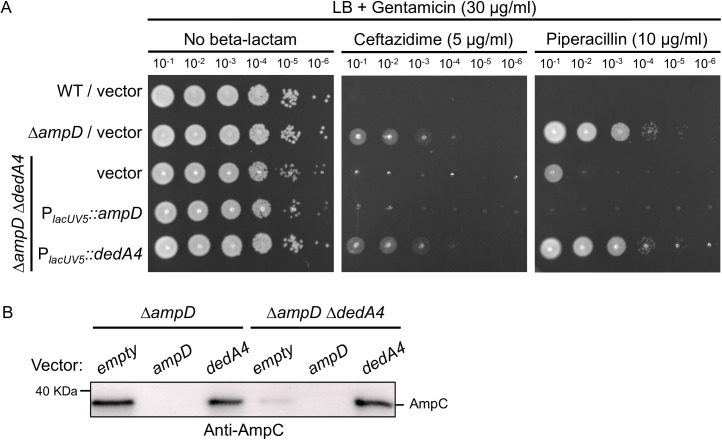
Expression of *dedA4* from a replicative plasmid restores AmpC production ad beta-lactam resistance in ∆*ampD* ∆*dedA4* cells. **(A)** Cultures of strains PAO1 [WT], CF5 [∆*ampD*] and CF1844 [∆*ampD* ∆*dedA4*] containing plasmids pPSV38 [vector control], pCF1098 [P_*lacUV5*_::*ampD*], or pCF835 [P_*lacUV5*_::*dedA4*] were serially diluted and 5 µl of each dilution was spotted onto LB agar supplemented with gentamicin (30 µg/ml) for plasmid maintenance, with ceftazidime (5 µg/ml) or piperacillin (10 µg/ml), as indicated. Plates were incubated overnight at 30°C and photographed. **(B)** Immunoblot for AmpC protein using the strains from panel (A).

To determine whether DedA4 influences beta-lactam resistance through AmpC, we quantified AmpC production and activity using Western blot and nitrocefin hydrolysis assays. As expected, the production of AmpC was undetectable in the wild-type, and ∆*dedA4* strains, while it strongly accumulated in the ∆*ampD* strains. However, the level produced was clearly reduced in the double mutants ∆*ampD* ∆*dedA4* (Fig 2C). Similar observations were made for AmpC activity. The level of nitrocefin hydrolysis detected for the ∆*ampD* lysates was high, with all the nitrocefin being hydrolyzed after 30 minutes of incubation, while the double mutant lysates still haven’t processed all the nitrocefin after 2 hours of incubation (Fig 2D). Notably, beta-lactam resistance and AmpC production were restored back to the level of the ∆*ampD* strain upon expression of *dedA4* from a plasmid in ∆*ampD* ∆*dedA4* cells (Fig 3A and 3B), indicating that the phenotype of the deletion allele was caused by the inactivation of DedA4 and not a polar effect of the deletion on the expression of the nearby genes. Expression of *ampD* in ∆*ampD* ∆*dedA4* cells from the same plasmid reversed the derepression, restoring beta-lactam sensitivity and eliminating AmpC production (Fig 3A and 3B). Collectively, these results support the idea that DedA4 contributes to beta-lactam resistance by modulating *ampC* induction, likely through its role in UndP recycling (Fig 1C).

### UndP flippase activity is required for full *ampC* induction and beta-lactam resistance in ∆*ampD* mutants

**Fig 4:**
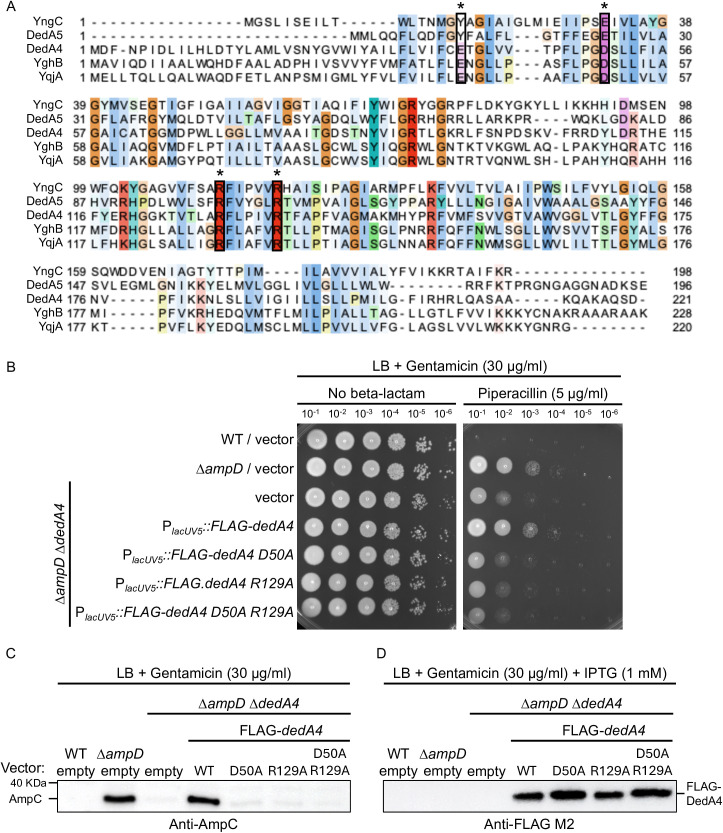
UndP recycling activity of DedA4 is required for complementation of ∆*ampD* ∆*dedA4* mutant. **(A)** Multiple sequence alignment (ClustalW) of DedA homologues (*B. subtilis* YngC; *P. aeruginosa* DedA4 and DedA5; *E. coli* YghB and YqjA) [28]. Four highly conserved residues are boxed in black and marked with an asterisk (see text for details). Cultures of strains PAO1 [WT], CF5 [∆*ampD*] and CF1844 [∆*ampD* ∆*dedA4*] carrying plasmids pPSV38 [vector control], pCF661 [P_*lacUV5*_::FLAG-*dedA4*], pCF580 [P_*lacUV5*_::FLAG-*dedA4 D50A*], pCF584 [P_*lacUV5*_::FLAG-*dedA4 R129A*] or pCF1154 [P_*lacUV5*_::FLAG-*dedA4 D50A R129A*] were serially diluted and 5 µl of each dilution was spotted onto LB agar supplemented with gentamicin (30 µg/ml) for plasmid maintenance, with or without piperacillin (5 µg/ml), as indicated. (**B**) Immunoblot for AmpC protein using the strains from panel (A). (**C**) Immunoblot for FLAG-tagged DedA4 variants using the same strains as in panel (A) grown for four hours in LB supplemented with gentamicin (30 µg/ml) and IPTG (1 mM).

It was previously demonstrated that DedA4 was able to complement an *uptA ykoX* double mutant in *B. subtilis* that lacks both genes encoding UndP flippases [20]. ClustalW alignment of *B. subtilis* UptA (YngC), *E. coli* YqjA and YghB and *P. aeruginosa* DedA4 and DedA5 highlighted the presence of conserved and essential residues for their function. DedA4^*Pae*^ Glu38 and Asp50 likely bind protons, while Arg129 and Arg135 likely bind the negatively charged phosphate group of UndP (Fig 4A) [20,26–28]. To test if UndP transport activity was necessary for *ampC* induction in the ∆*ampD* strain, we produced a FLAG-tagged variant of either DedA4(WT) or alleles mutated for two of these key residues. ∆*ampD* ∆*dedA4* cells expressing the functional mutants DedA4(D50A), DedA4(R129A) and the double mutant DedA4(D50A, R129A), failed to restore beta-lactam resistance and AmpC production, unlike cells expressing the WT DedA allele (Fig 4B and 4C). These results indicate that DedA4 function as an UndP flippase is required for optimal *ampC* induction in the ∆*ampD* strain. All mutant variants were produced at similar levels upon IPTG (1mM) treatment (Fig 4D).

Since *P. aeruginosa* genome encodes five DedA paralogues [25], we attempted to complement the ∆*ampD* ∆*dedA4* mutant with each of them. Complementation only worked with *dedA4* and *dedA5* when IPTG was added (S1A and S1B Fig). These results suggest that DedA4 is the major DedA family member involved in UndP recycling under our conditions. Although *dedA5* deletion in the ∆*ampD* strain had minimal impact on beta-lactam resistance (S2 Fig), overexpression of *dedA5* with IPTG restored AmpC production in the ∆*ampD* ∆*dedA4* background (S1B Fig). This suggests that DedA5 may primarily transport a different anionic lipid or may function under distinct conditions but can substitute for UndP flippase activity when overexpressed, similar to YkoX in *B. subtilis* [20]. Overexpression of *dedA3* with IPTG may slightly restore AmpC production and beta-lactam resistance in the ∆*ampD* ∆*dedA4* background (S1A and S1B Fig). Interestingly, overexpression of *dedA4* appears detrimental to the ∆*ampD* ∆*dedA4* strain. This is evidenced by the formation of smaller colonies on IPTG-containing plates (S1A Fig) and complete growth inhibition in the presence of both IPTG and piperacillin (S1A Fig, right panel), despite continued high-level AmpC production (S1B Fig, bottom panel). These findings suggest that overexpression of DedA4 may have toxic effects independent of *ampC* regulation. One possibility is that excess DedA4 perturbs membrane homeostasis by acting on lipids other than UndP. Supporting this idea, other DedA family members have been implicated in the transport of diverse lipids. For example, *B. subtilis* PetA was shown to transport phosphatidylethanolamine [29].

**Fig 5:**
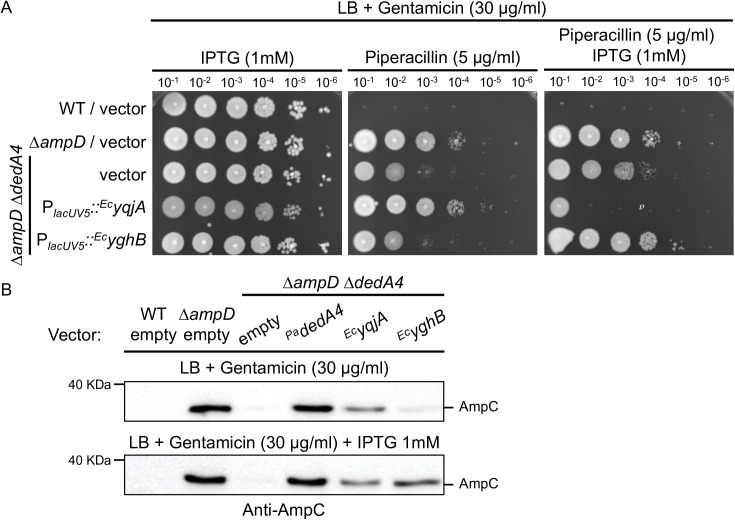
Heterologous complementation with DedA homologues from *E. coli* can complement ∆*ampD* ∆*dedA4.* **(A)** Cultures of strains PAO1 [WT], CF5 [∆*ampD*] and CF1844 [∆*ampD* ∆*dedA4*] carrying plasmids pPSV38 [vector control], pCF1147 [P_*lacUV5*_::^_*EcyqjA*_^] and pCF1150 [P_*lacUV5*_::^_*Ec*_^_*yghB*_] were serially diluted and 5 µl of each dilution was spotted onto LB agar supplemented with gentamicin (30 µg/ml) for plasmid maintenance, with or without piperacillin (5 µg/ml) or IPTG (1 mM), as indicated. **(B)** Immunoblot for AmpC protein using the strains from panel (A) grown in LB supplemented with gentamicin (30 µg/ml) and with or without IPTG (1 mM), as indicated.

Heterologous complementation with the UndP transporters of *E. coli* YqjA and YghB (homologues of DedA proteins), restore complete beta-lactam resistance and AmpC production to the ∆*ampD* ∆*dedA4* strain (Fig 5A and 5B). We note that overexpression of ^*Ec*^*yghB* with IPTG was required, like what we observed for ^*Pa*^*dedA5* (Fig 5A and 5B). This finding suggests that functional UndP flippase activity, regardless of its origin, is sufficient to restore *ampC* regulation in *P. aeruginosa*. We hypothesize that enough UndP lipid carriers on the inner leaflet of the cytoplasmic membrane is required to form lipid I and therefore reduced the pool of soluble PG precursors in the cytoplasm. In the absence of UndP recycling, soluble PG precursors may accumulate in the cytoplasm, bind to AmpR and maintain it in a repressive state, thereby limiting *ampC* induction.

### Disruption of UndP recycling alters the ratio of PG recycling products to PG precursors and suppresses *ampC* induction

The results presented thus far suggest that mutants lacking DedA4 are impaired in UndP recycling and accumulate cytoplasmic PG precursors (UDP-MurNAc-pentapeptide, UDP-M5). To test this hypothesis, we first performed muropeptide analysis to determine whether the overall structure of PG was altered in cells lacking *dedA4*. Consistent with prior results obtained in *V. cholerae* lacking *vca0040*, the *dedA4* mutant derivatives had 10% to 20% less PG than the wild-type and minor cross-linking defects (S3A-S3C Fig) [19]. We then quantified both PG recycling products including anhydro-muramyl tripeptide (anhMP-P3) and pentapeptide (anhMP-P5), along with the soluble PG precursor (UDP-M5) across different mutant backgrounds.

**Fig 6:**
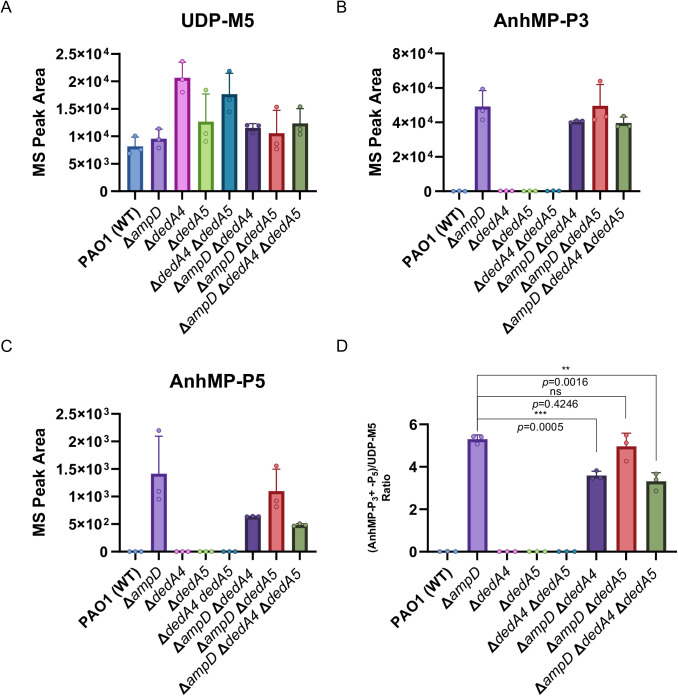
Changes in the balance of anhMP-P3 + anhMP-P5/ PG precursor in ∆*ampD* ∆*dedA4* confirmed by soluble muropeptide analyses. (**A-C**) Quantification of UDP-MurNAC pentapeptide (UDP-M5) (**A**), tripeptide-anhMP (anhMP-P3) (**B**) and pentapeptide-anhMP (anhMP-P5) (**C**) in strains PAO1 [WT], CF5 [∆*ampD*], CF1842 [∆*dedA4*], CF2034 [∆*dedA5*], CF2037 [∆*dedA4* ∆*dedA5*], CF1844 [∆*ampD* ∆*dedA4*], CF2041 [∆*ampD* ∆*dedA5*] and CF2043 [∆*ampD* ∆*dedA4* ∆*dedA5*]. (**D**) Ratio of PG recycling products (tripeptide-anhMP and pentapeptide-anhMP) to PG precursors (UDP-M5) in the same strains.

PG precursor levels were elevated in the ∆*dedA4* mutant (Fig 6A), a phenotype which was also observed in *V. cholerae* and *S. aureus* lacking the UndP transporters Vca0040 and SAOUHSC_00846*,* respectively [19], suggesting DedA4 plays a role in lipid carrier recycling. Although the accumulation of PG precursors persisted in the ∆*ampD* ∆*dedA4* and ∆*ampD* ∆*dedA4* ∆*dedA5* strains, it was less pronounced than in the ∆*dedA4* single mutants and not significant (Fig 6A). As expected, anhMP-P3 accumulated in all ∆*ampD* derivatives, though it showed a modest decrease in the ∆*ampD* ∆*dedA4* and ∆*ampD* ∆*dedA4* ∆*dedA5* strains (Fig 6B). A similar trend was observed for anhMP-P5 (Fig 6C). The raw data used to create these graphs can be found in S4 Fig.

**Fig 7:**
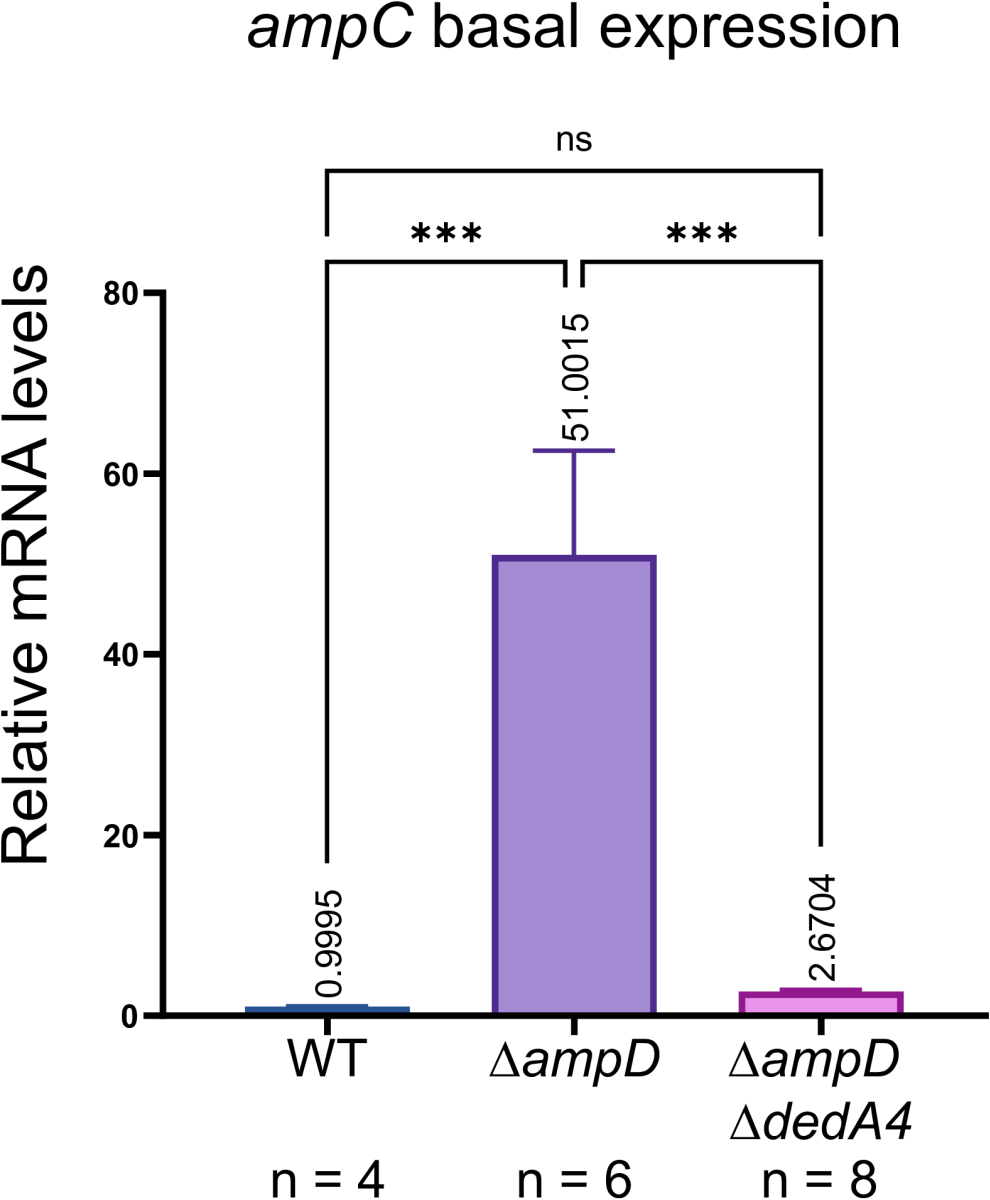
The change in anhMP-P3 + anhMP-P5/ UDP-M5 ratio is sufficient to switch most AmpR proteins to their repressive form. Basal expression of *ampC* in PAO1 [WT], CF5 [∆*ampD*] and CF1844 [∆*ampD* ∆*dedA4*].

Since anhMP-P5 and anhMP-P3 act as inducers and UDP-M5 as a repressor of *ampC* expression, we calculated their ratio to evaluate DedA’s role in maintaining their balance and, ultimately, its impact on beta-lactamase induction. Interestingly, the ratio of tri- and pentapeptide-anhMP to UDP-MurNAc-pentapeptides decreased significantly by 32% in the ∆*ampD* ∆*dedA4* strains and by 38% in the ∆*ampD* ∆*dedA4* ∆*dedA5* strains (Fig 6D) suggesting that AmpR shifted towards its repressor state. To confirm this, we measured *ampC* transcript levels using *rpsL* and *gyrB* as housekeeping controls. Expression of *ampC* was reduced 19-fold in the ∆*ampD* ∆*dedA4* double mutant, compared to the ∆*ampD* strain, which displays a strong *ampC* induction (51-fold increase relative to the wild-type) (Fig 7A). Taken together, these data confirm that deletion of *dedA4* in the ∆*ampD* mutant alters the intracellular ratio of PG precursors to recycling products, shifting AmpR activity toward its repressor form. This ultimately results in lower AmpC production and restores beta-lactam susceptibility in otherwise resistant ∆*ampD* strains (Fig 1C).

### Changing levels of UndP or PG precursors by expressing *uppS* or *murA* influence the level of beta-lactam resistance

**Fig 8:**
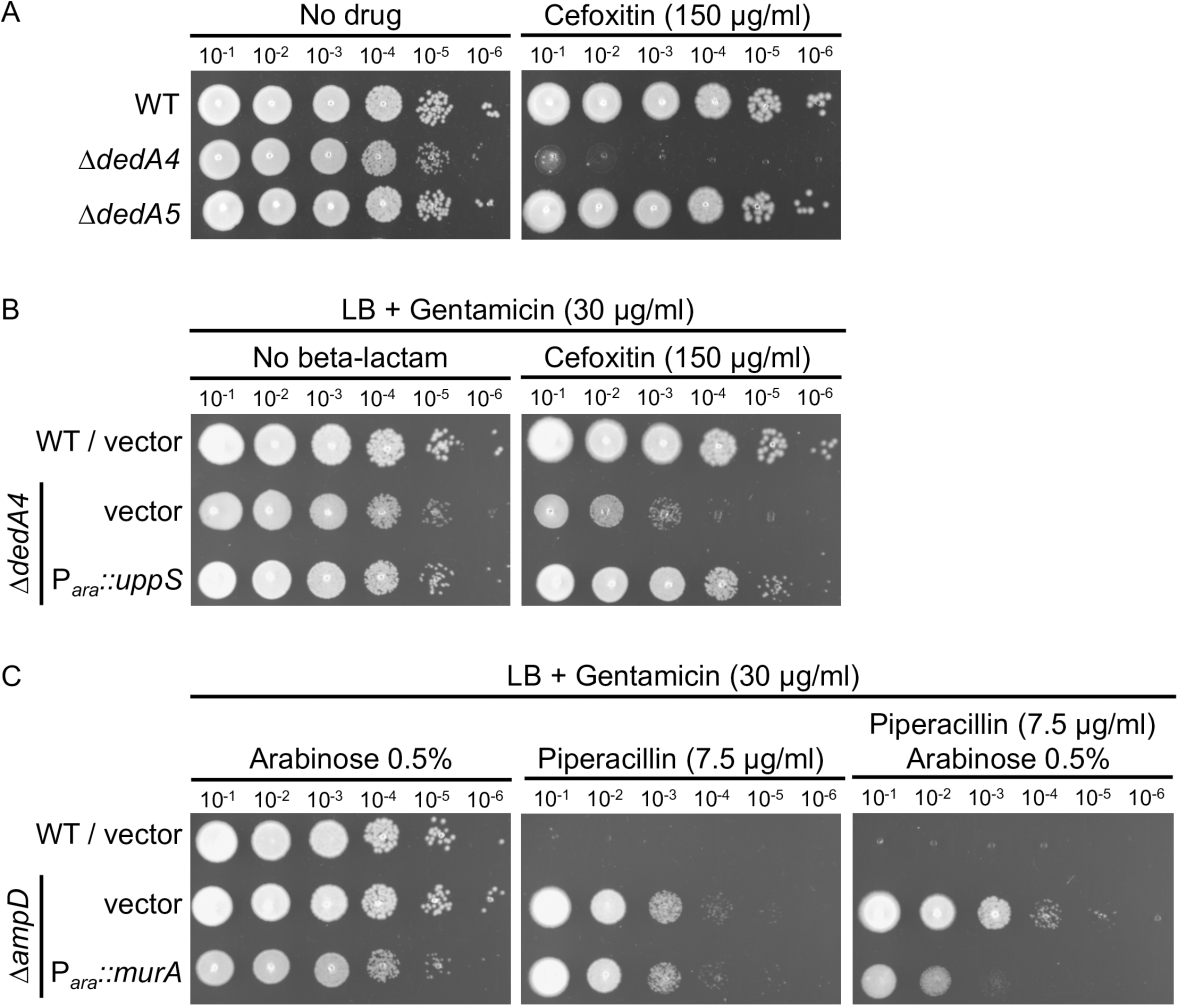
Increasing UndP or UDP-M5 levels by overproducing UppS or MurA, respectively, affects beta-lactam resistance, probably by modulating *ampC* induction. **(A)** Cultures of strain PAO1 [WT], CF1842 [∆*dedA4*] and CF2034 [∆*dedA5*] were serially diluted and 5 µl of each dilution was spotted onto LB agar supplemented with cefoxitin (150 µg/ml). **(B)**. Cultures of strain PAO1 [WT] and CF1842 [∆*dedA4*] with plasmids pJN105 [vector control] and pCF214 [P_*ara*_::*uppS*] were serially diluted and 5 µl of each dilution was spotted onto LB agar supplemented with gentamicin (30 µg/ml) for plasmid maintenance and cefoxitin (150 µg/ml), as indicated. **(C)** Cultures of strain PAO1 [WT] and CF5 [∆*ampD*] with plasmids pJN105 [vector control] and pCF166 [P_*ara*_::*murA*] were serially diluted and 5 µl of each dilution was spotted onto LB agar supplemented with gentamicin (30 µg/ml) for plasmid maintenance, arabinose (0.5%) and piperacillin (7.5 µg/ml), as indicated.

The ∆*dedA4* strain accumulates soluble PG precursors (UDP-M5) in its cytoplasm (Fig 6A). We hypothesized that this accumulation may interfere with AmpR activation during cefoxitin exposure, thereby rendering the ∆*dedA4* strain more sensitive to cefoxitin than the wild-type. Wild-type *P. aeruginosa* strains are typically highly resistant to cefoxitin, as it is both a potent *ampC* inducer and an effective AmpC substrate [4]. Indeed, we found that the ∆*dedA4* mutant was more susceptible to cefoxitin than both the wild-type and ∆*dedA5* strains, which display similar resistance profiles (Fig 8A). We next examined whether overexpressing *uppS* (*PA3652*), which encodes a key enzyme in the *de novo* synthesis of UndP, could restore cefoxitin resistance in the ∆*dedA4* background [21]. By increasing the supply of UndP lipid carriers, PG precursors can be more efficiently used for cell wall synthesis, reducing their cytoplasmic accumulation and relieving AmpR repression. As anticipated, *uppS* expression successfully restored the growth of the ∆*dedA4* strain to wild-type levels on plates containing 150 µg/ml cefoxitin (Fig 8B). In contrast, increasing the intracellular concentration of UDP-M5 in a ∆*ampD* mutant, achieved by overexpressing *murA* (*PA4450*), the enzyme catalyzing the first committed step in PG precursor biosynthesis, led to heightened sensitivity to beta-lactam antibiotics [30] (Fig 8C). Collectively, these findings emphasize the essential role of UndP availability in balancing PG precursors and regulating AmpC-mediated beta-lactam resistance, highlighting PG lipid carrier metabolism as a key factor in antibiotic susceptibility.

## Discussion

Overproduction of the beta-lactamase AmpC is a major resistance mechanism against beta-lactam antibiotics in *P. aeruginosa* and members of the Enterobacteriaceae, such as *Citrobacter freundii* and *Enterobacter cloacae* [4]. In these species, loss-of-function mutations in the cytoplasmic PG recycling amidase AmpD are a common route to constitutive *ampC* expression. [5,31–35]. In such mutants, PG recycling intermediates, particularly anhMP-P3, are thought to accumulate and to competitively displace the PG precursor bound to the transcriptional regulator AmpR, switching it to its activator state [14,15].

Several strategies have been explored to circumvent AmpC-mediated beta-lactam resistance. The most established approach involves combining beta-lactams with beta-lactamase inhibitors (BLIs). While piperacillin-tazobactam was widely used against *P. aeruginosa* [36], resistance has prompted the development of newer BLI combinations, such as ceftazidime-avibactam, imipenem-relebactam, and meropenem-vaborbactam [37]. These combinations improve efficacy but are still susceptible to resistance evolution [38].

Alternative strategies focus on disrupting *ampC* regulation. Inhibiting the permease AmpG prevents the uptake of anhydromuropeptides that signal AmpR activation [39,40], while blocking the glucosaminidase NagZ interferes with the generation of strong AmpR inducers [41]. Deletion of either gene or chemical inhibition (e.g., CCCP for AmpG or EtBuPUG for NagZ) reduces *ampC* expression and beta-lactam resistance in derepressed backgrounds [39–42].

Our work shows that PG precursor accumulation can be harnessed to suppress AmpC production, offering a new avenue for regulating antibiotic resistance (Fig 7B). Unlike conventional strategies that inhibit inducer production, this approach indirectly drives AmpR toward its repressor state by shifting precursor dynamics, unveiling a previously overlooked mechanism with significant therapeutic promise.

To experimentally test this concept, we targeted UndP recycling to manipulate PG precursor flux. Specifically, we deleted the gene encoding *P. aeruginosa* UndP flippase, *dedA4*, in an *ampC*-derepressed ∆*ampD* background to disrupt precursor utilization and promote their cytoplasmic accumulation. This deletion prevented AmpC production, AmpC activity and beta-lactam resistance (Figs 2 and 3). The UndP recycling activity of DedA4 was required to restore AmpC production in the ∆*ampD* ∆*dedA4* strain (Fig 4), and heterologous complementation with the *E. coli* UndP transporters YqjA and YghB could also complement the double mutant (Fig 5). We confirmed that the *dedA4* mutant had increased levels of soluble PG precursors, similar to *V. cholerae* and *S. aureus* strains deleted for DUF368 homologs (Fig 6A) [19]. Although PG precursor levels were not as elevated in the ∆*ampD* ∆*dedA4* strain, likely due to impaired precursor formation via the AmgK-MurU-MupP recycling pathway [43–46], the ratio of PG precursors to recycling products was significantly altered (Fig 6D). The modest changes observed in PG precursor and anhMP levels suggest that only slight fluctuations are sufficient to bias AmpR toward its repressor conformation. This finding highlights the requirement for a finely tuned balance of inducing molecules to fully activate AmpR. However, *in vivo* biochemical data on the binding affinities of AmpR for PG precursors or anhMP fragments remain scarce, limiting mechanistic interpretation. Despite this limitation, the observed shift was sufficient to favor repression, resulting in a 19-fold decrease in *ampC* transcript levels (Fig 7).Similarly, increasing PG precursor synthesis via *murA* overexpression increases antibiotic susceptibility of the *ampD* mutant, further supporting the idea that shifting the balance towards the PG precursor interferes with AmpR activation (Fig 8).

Notably, a recent study has also shown that DedA4 is required for *ampC* induction and beta-lactam resistance in a clinical bloodstream isolate of *P. aeruginosa* harboring a *dacB* mutation (PBP4 G437D) and therefore also derepressed for *ampC* [47,48]. Importantly, these results highlight that *ampC* expression depends not simply on the presence of inducers but on the ratio between PG recycling products and biosynthetic intermediates. This underscores the sensitivity of AmpR to subtle shifts in intracellular PG metabolite pools and suggests that interfering with UndP recycling is a viable strategy to manipulate *ampC* expression without targeting the enzyme or its regulators directly.

Natural products that target the UndP cycle, such as amphomycin, friulimicin B and bacitracin, bind to outward-facing UndPP or UndP, but their activity is largely limited to Gram-positive bacteria due to poor outer membrane permeability in Gram-negative species [49–51]. Nonetheless, the development of smaller, membrane-permeable molecules with similar targets specificity could provide a viable strategy to reduce *ampC* expression and potentiate beta-lactam antibiotics in *P. aeruginosa* and other pathogens encoding an inducible *ampC*.

Intriguingly, other UndP-linked pathways may influence this regulatory system. For example, biosynthesis of O-antigen and capsule also depends on UndP [16]. Competition between these pathways and PG synthesis could conceivably modulate precursor pools and impact AmpR signaling. Preliminary evidence from a *P. aeruginosa mucD* mutant, known to accumulate alginate [52], suggests that redirecting UndP flux toward exopolysaccharide production may also repress *ampC* induction and impact beta-lactam resistance (S5A and S5B Fig). If validated, this would broaden the metabolic processes implicated in beta-lactam resistance and support the idea that PG precursor homeostasis is a central regulatory hub. We aim to explore this in future work.

In summary, our findings reveal that AmpC expression is controlled by a finely tuned balance between PG precursors and recycling products. Disruption of UndP recycling shifts this balance toward precursor accumulation, thereby repressing AmpR and *ampC* even in genetically derepressed backgrounds. This work identifies a novel strategy to suppress beta-lactam resistance and underscores the importance of lipid carrier dynamics in coordinating antibiotic response pathways. Notably, similar links between membrane homeostasis and resistance have been observed in *B. subtilis* and *Burkholderia thailandensis*, where mutations in *dedA* homologs modulate susceptibility to duramycin and colistin, respectively [29,53]. Targeting UndP recycling offers a promising new direction for adjunctive therapies that could resensitize AmpC-producing bacteria to existing beta-lactam antibiotics.

## Methods and materials

### Media, bacterial strains and plasmids

*P. aeruginosa* PAO1 cells were grown in LB (1% tryptone, 0.5% yeast extract, 1% NaCl). As indicated, the medium was supplemented with 1 mM IPTG (isopropyl beta-D-1-thiogalactopyranoside) or 0.5% arabinose. For plasmid maintenance, gentamicin (Gm) was used at a concentration of 30 μg/mL. Unless otherwise indicated, antipseudomonal antibiotics for viability/sensitivity assays were used at 3, 5, 7.5 or 10 μg/mL (piperacillin; Pip or ceftazidime; Caz), as indicated. All *P. aeruginosa* strains used in the reported experiments are derivatives of PAO1. *E. coli* cells were grown in LB. For plasmid maintenance or selection in *E. coli*, antibiotic concentration used was 15 μg/mL (gentamicin; Gm). The bacterial strains and plasmids used in this study are listed in S1-S3 Tables. Detailed descriptions of the strain and plasmid construction procedures can be found in the S1 Appendix.

### *P. aeruginosa* viability assay

For viability assays, overnight cell cultures were normalized to an OD_600_ of 0.1 and subjected to serial 10-fold dilutions with LB. Five microliters of each dilution was then spotted onto the indicated agar and plates were incubated at 30°C for 24 hours prior to imaging.

### Growth curve

Strains were cultured overnight at 30°C in LB medium with agitation at 200 rpm. The bacterial cultures were then diluted to a final optical density at 600 nm (OD_600_) of 0.01 using the appropriate medium. The cultures were transferred to a 96-well plate and incubated with continuous orbital agitation (282 cpm) at 30°C for 24 hours. OD_600_ readings were taken every 15 minutes using a Biotek Epoch 2 spectrophotometer (Agilent, Santa Clara, California, USA) and analyzed with BioTek Gen5 software (Agilent, California, USA).

### *P. aeruginosa* electroporation

*P. aeruginosa* strains were made competent using previously described methods [54]. Briefly, 4 mL of overnight cultures grown at 37°C were centrifuged and washed twice with 1 mL 300 mM sucrose. Cell pellets were resuspended in 500 µL of 300 mM sucrose and 100 µL were used for electroporation. 1µL of replicative plasmid was used for the electroporation (Gene Pulser, Bio-Rad), using the following settings: 25 mF, 200 O, 2.5 kV. LB medium (1mL) was added and the cells incubated with shaking (200 rpm) for 1 h at 37°C. Cells were then plated on the appropriate selective medium.

### AmpC beta-lactamase activity assay

AmpC activity was assessed using nitrocefin hydrolysis. Overnight bacterial cultures were subcultured 1:20 in 3 mL LB and grown for 4 hr at 30°C and 200 rpm. Following incubation, 1 mL of culture was pelleted at 5’000 x g for 5 minutes, washed once with 1 mL of 50 mM sodium phosphate buffer (pH 7.0) and resuspended in 2 mL of the same cold buffer. The samples were frozen on dry ice for 15 minutes and thawed on ice. Samples were placed on ice and lysed at 4°C by sonication with a microprobe (Q800R2, QSonica, Newtown, Connecticut, USA). Sonicated samples were centrifuged at 12,000 x g for 10 minutes at 4°C and supernatants were collected. The protein concentration was determined using a Bradford assay [55] with bovine serum albumin (BSA) as the standard (G-Biosciences, Geno technology inc., Saint-Louis, Missouri, USA). Nitrocefin hydrolysis assays were performed in 96-well plates. Each reaction had a final volume of 250 μl of 50 mM sodium phosphate buffer (pH 7.0) containing 10 μg of protein and 20 μg of nitrocefin (Thermo Fischer Scientific Oxoid, Waltham, Massachusetts, USA). Nitrocefin hydrolysis was monitored by measuring the absorbance at 340 and 486 nm every 2 minutes for 120 minutes at 30°C.

### Antibiotic sensitivity assays

Antibiotic sensitivity assays were performed using broth microdilutions. Overnight cell cultures were normalized to OD_600_ of 0.0005 in LB and the indicated concentrations of piperacillin, ceftazidime, A22, tobramycin or gentamicin, and grown for 24 hours at 30°C prior to taking optical density readings (Biotek Epoch 2, Agilent, Santa Clara, California, USA). Broth microdilution MIC assays were performed three or four times independently, each with three technical replicates.

### Intracellular soluble muropeptide analysis

To determine the presence and levels of intracellular soluble muropeptides, bacteria were grown until late exponential phase (roughly OD_600_ 0.7) in LB media before being cooled on ice for 10 min and normalized to the same OD_600_. Cells were then harvested by centrifugation at 10,000 × *g* for 10 min. The supernatant was discarded, and the cell pellet was washed three times in ice-cold 0.9% NaCl, resuspended in 0.9% NaCl so that the cells are 20 times concentrated and boiled for 10 min before centrifugation at maximum speed in a benchtop centrifuge for 10 min to remove the proteins and insoluble fraction. The supernatant was used for further analysis by LC-MS.

### Peptidoglycan isolation

Cells from 0.2 L cultures of overnight stationary phase were pelleted at 5,250 x g and resuspended in 5 ml of PBS, added to an equal volume of 10% SDS in a boiling water bath and vigorously stirred for 3 h, then stirred overnight at room temperature. The insoluble fraction (peptidoglycan) was pelleted at 400,000 x g, 15 min, 30 ºC (TLA-100.3 rotor; Optima Max ultracentrifuge, Beckman) and resuspended in Milli-Q water. This step was repeated 4–5 times until the SDS was washed out. Next, peptidoglycan was treated with proteinase K (40 μg/ml in 100 323 mM TrisHCl pH 8.0), 30 min at 37 ºC and then boiled in 1% SDS for 2 h to stop the reaction. After SDS was removed as described previously, peptidoglycan samples were resuspended in 200 μL of 50 mM sodium phosphate buffer pH 4.9 and digested overnight with 30 μg/ml muramidase (from *Streptomyces albus*) at 37 ºC. Muramidase digestion was stopped by heat-inactivation (boiling during 5 min). Coagulated protein was removed by centrifugation (20,000 x g, 15 min). The supernatants (soluble muropeptides) were subjected to sample reduction. First, pH was adjusted to 8.5-9 by addition of borate buffer 0.5 M pH 9 and then muramic acid residues were reduced by sodium borohydride treatment (NaBH_4_ 10 mg/ml final concentration) during 30 min at room temperature. Finally, pH was adjusted to 2.0-4.0 with orthophosphoric acid 25% prior to analysis by LC.

### LC-MS analysis

Chromatographic analyses of muropeptides were performed by Ultra Performance Liquid Chromatography (UPLC) on an UPLC system (Waters) equipped with a trapping cartridge precolumn (SecurityGuard ULTRA Cartridge UHPLC C18 2.1 mm, Phenomenex) and an analytical column BEH C18 column (130 Å, 1.7 μm, 2.1 mm, Waters) maintained at 45°C. Muropeptides were detected by measuring the absorbance at 204 nm using an ACQUITY UPLC UV−visible Detector. Muropeptides were separated using a linear gradient from buffer A (Water + 0.1% (v/v) formic acid) to buffer B (Acetonitrile 100% (v/v) + 0.1% (v/v) formic acid) over 15 min with a flowrate of 0.25 ml/min. The QTOF instrument was operated in positive ion mode, with data collection performed in untargeted MS^e^ mode. The parameters were set as follows: capillary voltage 3.0 kV, source temperature 120°C, desolvation temperature 350°C, sample cone voltage 40 V, cone gas flow 100 L h^−1^ and desolvation gas flow 500 L h^−1^. Mass spectra were acquired at a speed of 0.25 s/scan. The scan was in a range of 100–2000 m/z. Data acquisition and processing was performed using MassLynx or UNIFI software package (Waters Corp.). The quantification of muropeptides was based on their relative abundances (relative area of the corresponding peak) and relative molar abundances. A table of all the identified muropeptides and the observed ions is provided (S4 Table). The raw data used to create these graphs can be found in S4 Fig.

### Statistical analysis and reproducibility

Statistical evaluations were conducted using Prism 8.0 (GraphPad Software, USA). For comparisons, two-tailed unpaired t-tests were applied. A p-value threshold of less than 0.05 was considered statistically significant, with significance levels denoted as follows: *p* < 0.05, *p* < 0.01, *p < 0.001, and **p < 0.0001. All assays incorporated appropriate control groups, and both experimental and control conditions were carried out using isogenic strains. Experiments included a minimum of three independent biological replicates.

### Immunoblotting

Overnight bacterial cultures were subcultured 1:100 in 3 mL of appropriate medium and grown for 4 hours. Bacteria were normalized to an OD_600nm_ of 0.5 in 1 ml, pelleted and resuspended in a final volume of 100 µl. 20 µl of Laemmli buffer were added and the samples were then boiled for 10 minutes at 95°C. Immunoblotting was performed by first separating 10 µL of each sample on 12% SDS-PAGE (polyacrylamide gel electrophoresis) gels at 70V for 30 minutes and 120V for an hour. Proteins were transferred at 90V for an hour to a 0.2 μm PolyVinylidene DiFluoride (PVDF) membranes (Immobilon-FL PVDF membrane, IPFL00010, Merck) previously soaked in methanol and rinsed with transfer buffer. Membranes were stained with Ponceau S solution to ensure equal loading in each lane and transfer. Membranes were blocked using 5% (w/v) skim milk in Tris-Buffered Saline (10 mM Tris-HCl pH 7.5, 150 mM NaCl) supplemented with 0.1% (v/v) Tween-20 (TBS-T) for 1 hour. Membranes were incubated for 1 hour with α-AmpC primary antibody (1:1000 dilution in 5% skim milk in TBS-T, MyBioSource, MBS1493275, San Diego, USA) or α-FLAG primary antibody (1:1000 dilution in 5% skim milk in TBS-T, F7425, Merck). The membranes were washed four times in TBS-T for 5 min each before incubation for 1 h with secondary antibody (anti-rabbit IgG HRP, 1: 10000 dilution, A0545, Merck) in TBS-T with 5% (w/v) skim milk powder. The membranes were then washed four times with TBS-T for 5 min each before developing using Immobilon Western Chemiluminescent HRP Substrate (WBKLS0100, Merck) and imaged using the FUSION FX Spectra imaging platform (VILBER). Images were acquired with the Evolution Cap Edge software.

### RT-qPCR

The relative expression level of *ampC* was determined by quantitative reverse transcription PCR (RT-qPCR). Total RNA was isolated from 1 ml of bacterial cultures in the logarithmic growth phase using the RNeasy 96 QIAcube HT Kit (Qiagen) according to the manufacturer’s instructions. One microgram of total RNA from each sample was reverse transcribed using the QuantiTect Reverse Transcription Kit (Qiagen) with random hexamers. The resulting cDNA was diluted 1:5 and analyzed in duplicate for quantitative PCR with SYBR Green master mix (BIO-RAD) using the QuantStudio 3 Real-Time PCR System (Applied Biosystems).

The threshold cycle (Ct) for each sample, corresponding to the PCR cycle at which fluorescence exceeds a predefined threshold, was determined using the QuantStudio software. The *rpsL* and *gyrB* housekeeping genes were used as internal controls. The primers used are listed in S5 Table. The mean values of relative mRNA expression (calculated as 2^(−ΔΔC^_T_^)^ [56]) from two independent biological replicates and two technical replicates were considered for analysis. The wild-type strain was used as the control.

Primers efficiencies were assessed by generating a standard curve with serial dilutions of a cDNA template. Primers efficiencies were determined to be ≥ 90%, with no more than a 5% variation between each pair of primers. A final melting curve step was systematically included to verify the specificity of the amplification.

## Supporting information

S1 Fig Expression of the five ^*Pa*^DedA family members in *P. aeruginosa* strains and their effect on AmpC production. (A) Cultures of the strains PAO1 [WT], CF5 [∆*ampD*] and CF1844 [∆*ampD* ∆*dedA4*], harboring an empty vector (pSV38) or plasmids pCF1141 (P*lacUV5*::*dedA1*), pCF1145 (P*lacUV5*::*dedA2*), pCF1137 (P*lacUV5*::*dedA3*), pCF835 (P*lacUV5*::*dedA4*) or pCF577 (P*lacUV5*::*dedA5*) were diluted and 5 μl of each dilution was spotted onto LB agar supplemented with gentamicin 30 μg/ml for plasmid maintenance,with or without IPTG inducer and/or piperacillin (5 μg/ml). (B) Immunoblot for AmpC protein using the strains from panel (A) grown in LB supplemented with gentamicin (30 μg/ml) and with or without IPTG (1 mM), as indicated.
(TIF)

S2 Fig Beta-lactam susceptibility of strains with *dedA4* and *dedA5* deletions. Cultures of the strains PAO1 [WT], CF5 [∆*ampD*], CF1844 [∆*ampD* ∆*dedA4*], CF2041 [∆*ampD* ∆*dedA5*] and CF2043 [∆*ampD* ∆*dedA4* ∆*dedA5*] were diluted and 5 μl of each dilution was spotted onto LB agar supplemented with either ceftazidime (3 μg/ml) or piperacillin (5 μg/ml).
(TIF)

S3 Fig Peptidoglycan analysis and PG crosslinking in various strains. (A) Ultra Performance Liquid Chromatography (UPLC) PG analysis, with characteristic peaks labeled as follows: M = monomeric muropeptide (uncrosslinked), D = dimeric muropeptide (crosslink connecting two muropeptides), T = trimeric muropeptide (crosslinks connecting three muropeptides). Numbers indicate the status of the peptide side chain (3 = tripeptide, 4 = tetrapeptide). Strains used for analysis were: PAO1 [WT], CF5 [∆*ampD*], CF1842 [∆*dedA4*], CF2034 [∆*dedA5*], CF2037 [∆*dedA4* ∆*dedA5*], CF1844 [∆*ampD* ∆*dedA4*], CF2041 [∆*ampD* ∆*dedA5*] and CF2043 [∆*ampD* ∆*dedA4* ∆*dedA5*]. (B) Total peptidoglycan content in the same strains as in panel (A). (C) Analysis of PG crosslinking in the same strains as in panel (A).
(TIF)

S4 Fig Extracted ion chromatograms (XICs) of UDP-M5, anhMP3, and anhMP5 obtained by LC-MS. The ions were extracted at *m/z* 597.67 [M + 2H]2 + , 648.27 [M + H]+, and 790.34 [M + H]+, respectively. (TIF)

S5 Fig MucD is required for AmpC production and beta-lactam resistance in cells defective for AmpD. (A) Cultures of strains PAO1 [WT], CF482 [∆*mucD*], CF5 [∆*ampD*] and CF1805 [∆*ampD* ∆*mucD*] were serially diluted and 5 µl of each dilution was spotted onto LB agar supplemented with either ceftazidime (5 µg/ml) or piperacillin (10 µg/ml), as indicated. Plates were incubated overnight at 30^o^C and photographed. (B) Immunoblot for AmpC protein using the strains from panel (A).
(TIF)

S1 Table *Pseudomonas aeruginosa* strains used in this study. (DOCX)

S2 Table *Escherichia coli* strains used in this study. (DOCX)

S3 Table Plasmids used in this study. (DOCX)

S4 Table Identified muropeptides. (DOCX)

S5 Table Primers used for RT-qPCR. (DOCX)

S1 Appendix Strain and plasmid construction procedures. (DOCX)

## References

[1] Strateva T, Yordanov D. Pseudomonas aeruginosa - a phenomenon of bacterial resistance. J Med Microbiol. 2009;58(Pt 9):1133–48. doi: 10.1099/jmm.0.009142-0 19528173

[2] López-Causapé C, Cabot G, Del Barrio-Tofiño E, Oliver A. The Versatile Mutational Resistome of Pseudomonas aeruginosa. Front Microbiol. 2018;9:685. doi: 10.3389/fmicb.2018.00685 29681898 PMC5897538

[3] Livermore DM. beta-Lactamases in laboratory and clinical resistance. Clin Microbiol Rev. 1995;8(4):557–84. doi: 10.1128/CMR.8.4.557 8665470 PMC172876

[4] Jacoby GA. AmpC β-lactamases. Clinical Microbiology Reviews. 2009;22(1):161–82. 19136439 10.1128/CMR.00036-08 PMC2620637

[5] Fisher JF, Mobashery S. The sentinel role of peptidoglycan recycling in the β-lactam resistance of the Gram-negative Enterobacteriaceae and Pseudomonas aeruginosa. Bioorg Chem. 2014;56:41–8. doi: 10.1016/j.bioorg.2014.05.011 24955547 PMC4161644

[6] Livermore DM. Clinical significance of beta-lactamase induction and stable derepression in gram-negative rods. Eur J Clin Microbiol. 1987;6(4):439–45. doi: 10.1007/BF02013107 3311738

[7] Aguilera Rossi CG, Gómez-Puertas P, Ayala Serrano JA. In vivo functional and molecular characterization of the Penicillin-Binding Protein 4 (DacB) of Pseudomonas aeruginosa. BMC Microbiol. 2016;16(1):234. doi: 10.1186/s12866-016-0853-x 27716106 PMC5054556

[8] Höltje JV, Kopp U, Ursinus A, Wiedemann B. The negative regulator of beta-lactamase induction AmpD is a N-acetyl-anhydromuramyl-L-alanine amidase. FEMS Microbiol Lett. 1994;122(1–2):159–64. doi: 10.1111/j.1574-6968.1994.tb07159.x 7958768

[9] Boudreau MA, Fisher JF, Mobashery S. Messenger functions of the bacterial cell wall-derived muropeptides. Biochemistry. 2012;51(14):2974–90. doi: 10.1021/bi300174x 22409164 PMC3345243

[10] Lee M, Dhar S, De Benedetti S, Hesek D, Boggess B, Blázquez B, et al. Muropeptides in Pseudomonas aeruginosa and their Role as Elicitors of β-Lactam-Antibiotic Resistance. Angew Chem Int Ed Engl. 2016;55(24):6882–6. doi: 10.1002/anie.201601693 27111486 PMC5081488

[11] Dik DA, Domínguez-Gil T, Lee M, Hesek D, Byun B, Fishovitz J, et al. Muropeptide Binding and the X-ray Structure of the Effector Domain of the Transcriptional Regulator AmpR of Pseudomonas aeruginosa. J Am Chem Soc. 2017;139(4):1448–51. doi: 10.1021/jacs.6b12819 28079369 PMC5436579

[12] Vadlamani G, Thomas MD, Patel TR, Donald LJ, Reeve TM, Stetefeld J, et al. The β-lactamase gene regulator AmpR is a tetramer that recognizes and binds the D-Ala-D-Ala motif of its repressor UDP-N-acetylmuramic acid (MurNAc)-pentapeptide. J Biol Chem. 2015;290(5):2630–43. doi: 10.1074/jbc.M114.618199 25480792 PMC4316999

[13] Jacobs C, Frère JM, Normark S. Cytosolic intermediates for cell wall biosynthesis and degradation control inducible beta-lactam resistance in gram-negative bacteria. Cell. 1997;88(6):823–32. doi: 10.1016/s0092-8674(00)81928-5 9118225

[14] Torrens G, Hernández SB, Ayala JA, Moya B, Juan C, Cava F. Regulation of AmpC-Driven β-Lactam Resistance in Pseudomonas aeruginosa: Different Pathways, Different Signaling. mSystems. 2019;4(6). 10.1128/mSystems.00524-19 PMC6890930 31796566

[15] Jacobs C, Huang LJ, Bartowsky E, Normark S, Park JT. Bacterial cell wall recycling provides cytosolic muropeptides as effectors for beta-lactamase induction. EMBO J. 1994;13(19):4684–94. doi: 10.1002/j.1460-2075.1994.tb06792.x 7925310 PMC395403

[16] Manat G, Roure S, Auger R, Bouhss A, Barreteau H, Mengin-Lecreulx D, et al. Deciphering the metabolism of undecaprenyl-phosphate: the bacterial cell-wall unit carrier at the membrane frontier. Microb Drug Resist. 2014;20(3):199–214. doi: 10.1089/mdr.2014.0035 24799078 PMC4050452

[17] Rohs PDA, Bernhardt TG. Growth and Division of the Peptidoglycan Matrix. Annu Rev Microbiol. 2021;75:315–36. doi: 10.1146/annurev-micro-020518-120056 34351794

[18] Workman SD, Strynadka NCJ. A Slippery Scaffold: Synthesis and Recycling of the Bacterial Cell Wall Carrier Lipid. J Mol Biol. 2020;432(18):4964–82. doi: 10.1016/j.jmb.2020.03.025 32234311

[19] Sit B, Srisuknimit V, Bueno E, Zingl FG, Hullahalli K, Cava F, et al. Undecaprenyl phosphate translocases confer conditional microbial fitness. Nature. 2023;613(7945):721–8. doi: 10.1038/s41586-022-05569-1 36450355 PMC9876793

[20] Roney IJ, Rudner DZ. Two broadly conserved families of polyprenyl-phosphate transporters. Nature. 2023;613(7945):729–34. doi: 10.1038/s41586-022-05587-z 36450357 PMC10184681

[21] Apfel CM, Takács B, Fountoulakis M, Stieger M, Keck W. Use of genomics to identify bacterial undecaprenyl pyrophosphate synthetase: cloning, expression, and characterization of the essential uppS gene. Journal of Bacteriology. 1999;181(2):483–92. 9882662 10.1128/jb.181.2.483-492.1999 PMC93402

[22] Do T, Page JE, Walker S. Uncovering the activities, biological roles, and regulation of bacterial cell wall hydrolases and tailoring enzymes. J Biol Chem. 2020;295(10):3347–61. 31974163 10.1074/jbc.REV119.010155 PMC7062177

[23] Sham L-T, Butler EK, Lebar MD, Kahne D, Bernhardt TG, Ruiz N. Bacterial cell wall. MurJ is the flippase of lipid-linked precursors for peptidoglycan biogenesis. Science. 2014;345(6193):220–2. doi: 10.1126/science.1254522 25013077 PMC4163187

[24] Piepenbreier H, Diehl A, Fritz G. Minimal exposure of lipid II cycle intermediates triggers cell wall antibiotic resistance. Nat Commun. 2019;10(1):2733. doi: 10.1038/s41467-019-10673-4 31227716 PMC6588590

[25] Justice MR, Justice JS, Doerrler WT. The Conserved DedA/Tvp38 Membrane Protein Family Plays a Role in Antibiotic Resistance in Pseudomonas aeruginosa. The FASEB Journal. 2016;30(S1). doi: 10.1096/fasebj.30.1_supplement.853.1

[26] Todor H, Herrera N, Gross CA. Three Bacterial DedA Subfamilies with Distinct Functions and Phylogenetic Distribution. mBio. 2023;14(2):e0002823. doi: 10.1128/mbio.00028-23 36856409 PMC10127716

[27] Scarsbrook HL, Urban R, Streather BR, Moores A, Mulligan C. Topological analysis of a bacterial DedA protein associated with alkaline tolerance and antimicrobial resistance. Microbiology (Reading). 2021;167(12):10.1099/mic.0.001125. doi: 10.1099/mic.0.001125 34914576

[28] Larkin MA, Blackshields G, Brown NP, Chenna R, McGettigan PA, McWilliam H. Clustal W and Clustal X version 2.0. Bioinformatics. 2007;23(21):2947–8. 17846036 10.1093/bioinformatics/btm404

[29] Roney IJ, Rudner DZ. The DedA superfamily member PetA is required for the transbilayer distribution of phosphatidylethanolamine in bacterial membranes. Proc Natl Acad Sci U S A. 2023;120(20):e2301979120. doi: 10.1073/pnas.2301979120 37155911 PMC10193950

[30] Marquardt JL, Siegele DA, Kolter R, Walsh CT. Cloning and sequencing of Escherichia coli murZ and purification of its product, a UDP-N-acetylglucosamine enolpyruvyl transferase. J Bacteriol. 1992;174(17):5748–52. doi: 10.1128/jb.174.17.5748-5752.1992 1512209 PMC206525

[31] Bagge N, Ciofu O, Hentzer M, Joan I a, Givskov M, Høiby N, et al. Constitutive high expression of chromosomal β-lactamase in Pseudomonas aeruginosa caused by a new insertion sequence (IS 1669) located in ampD. Antimicrobial Agents and Chemotherapy. 2002;46(11):3406–12. 12384343 10.1128/AAC.46.11.3406-3411.2002 PMC128714

[32] Stapleton P, Shannon K, Phillips I. DNA sequence differences of ampD mutants of Citrobacter freundii. Antimicrob Agents Chemother. 1995;39(11):2494–8. doi: 10.1128/AAC.39.11.2494 8585732 PMC162971

[33] Kopp U, Wiedemann B, Lindquist S, Normark S. Sequences of wild-type and mutant ampD genes of Citrobacter freundii and Enterobacter cloacae. Antimicrob Agents Chemother. 1993;37(2):224–8. doi: 10.1128/AAC.37.2.224 8383940 PMC187643

[34] Korfmann G, Sanders CC, Moland ES. Altered phenotypes associated with ampD mutations in Enterobacter cloacae. Antimicrob Agents Chemother. 1991;35(2):358–64. doi: 10.1128/AAC.35.2.358 2024967 PMC245005

[35] Lindberg F, Westman L, Normark S. Regulatory components in Citrobacter freundii ampC beta-lactamase induction. Proc Natl Acad Sci U S A. 1985;82(14):4620–4. doi: 10.1073/pnas.82.14.4620 2991883 PMC390437

[36] Lodise TP Jr, Lomaestro B, Drusano GL. Piperacillin-tazobactam for Pseudomonas aeruginosa infection: clinical implications of an extended-infusion dosing strategy. Clin Infect Dis. 2007;44(3):357–63. doi: 10.1086/510590 17205441

[37] Le Terrier C, Raro OHF, Saad AM, Nordmann P, Poirel L. In-vitro activity of newly-developed β-lactamase inhibitors avibactam, relebactam and vaborbactam in combination with anti-pseudomonal β-lactam antibiotics against AmpC-overproducing clinical Pseudomonas aeruginosa isolates. Eur J Clin Microbiol Infect Dis. 2025;44(2):277–84. 39589655 10.1007/s10096-024-04965-x PMC11754317

[38] Papp-Wallace KM, Mack AR, Taracila MA, Bonomo RA. Resistance to Novel β-Lactam-β-Lactamase Inhibitor Combinations: The “Price of Progress”. Infect Dis Clin North Am. 2020;34(4):773–819. doi: 10.1016/j.idc.2020.05.001 33011051 PMC7609624

[39] Zamorano L, Reeve TM, Juan C, Moya B, Cabot G, Vocadlo DJ. AmpG inactivation restores susceptibility of pan-lactam-resistant Pseudomonas aeruginosa clinical strains. Antimicrobial Agents and Chemotherapy. 2011;55(5):1990–6. 21357303 10.1128/AAC.01688-10 PMC3088256

[40] Zhang Y, Bao Q, Gagnon LA, Huletsky A, Oliver A, Jin S, et al. ampG gene of Pseudomonas aeruginosa and its role in β-lactamase expression. Antimicrob Agents Chemother. 2010;54(11):4772–9. doi: 10.1128/AAC.00009-10 20713660 PMC2976151

[41] Zamorano L, Reeve TM, Deng L, Juan C, Moyá B, Cabot G. NagZ inactivation prevents and reverts β-lactam resistance, driven by AmpD and PBP 4 mutations, in Pseudomonas aeruginosa. Antimicrobial Agents and Chemotherapy. 2010;54(9):3557–63. 20566764 10.1128/AAC.00385-10 PMC2934985

[42] Asgarali A, Stubbs KA, Oliver A, Vocadlo DJ, Mark BL. Inactivation of the glycoside hydrolase NagZ attenuates antipseudomonal beta-lactam resistance in Pseudomonas aeruginosa. Antimicrob Agents Chemother. 2009;53(6):2274–82. doi: 10.1128/AAC.01617-08 19273679 PMC2687237

[43] Fumeaux C, Bernhardt TG. Identification of mupP as a new peptidoglycan recycling factor and antibiotic resistance determinant in Pseudomonas aeruginosa. mBio. 2017;8(2). 10.1128/mBio.00102-17 PMC5371409 28351916

[44] Borisova M, Gisin J, Mayer C. The N-Acetylmuramic Acid 6-Phosphate Phosphatase MupP Completes the Pseudomonas Peptidoglycan Recycling Pathway Leading to Intrinsic Fosfomycin Resistance. mBio. 2017;8(2):e00092-17. doi: 10.1128/mBio.00092-17 28351914 PMC5371407

[45] Borisova M, Gisin J, Mayer C. Blocking peptidoglycan recycling in Pseudomonas aeruginosa attenuates intrinsic resistance to fosfomycin. Microb Drug Resist. 2014;20(3):231–7. doi: 10.1089/mdr.2014.0036 24819062 PMC4050453

[46] Gisin J, Schneider A, Nägele B, Borisova M, Mayer C. A cell wall recycling shortcut that bypasses peptidoglycan de novo biosynthesis. Nat Chem Biol. 2013;9(8):491–3. doi: 10.1038/nchembio.1289 23831760

[47] Sonnabend MS, Klein K, Beier S, Angelov A, Kluj R, Mayer C, et al. Identification of Drug Resistance Determinants in a Clinical Isolate of Pseudomonas aeruginosa by High-Density Transposon Mutagenesis. Antimicrob Agents Chemother. 2020;64(3):e01771-19. doi: 10.1128/AAC.01771-19 31818817 PMC7038268

[48] Moya B, Dötsch A, Juan C, Blázquez J, Zamorano L, Haussler S, et al. Beta-lactam resistance response triggered by inactivation of a nonessential penicillin-binding protein. PLoS Pathog. 2009;5(3):e1000353. doi: 10.1371/journal.ppat.1000353 19325877 PMC2654508

[49] Storm DR, Strominger JL. Complex formation between bacitracin peptides and isoprenyl pyrophosphates. The specificity of lipid-peptide interactions. J Biol Chem. 1973;248(11):3940–5. 4350651

[50] Tanaka H, Iwai Y, Oiwa R, Shinohara S, Shimizu S, Oka T, et al. Studies on bacterial cell wall inhibitors. II. Inhibition of peptidoglycan synthesis in vivo and in vitro by amphomycin. Biochim Biophys Acta. 1977;497(3):633–40. doi: 10.1016/0304-4165(77)90283-5 407940

[51] Schneider T, Gries K, Josten M, Wiedemann I, Pelzer S, Labischinski H, et al. The lipopeptide antibiotic Friulimicin B inhibits cell wall biosynthesis through complex formation with bactoprenol phosphate. Antimicrob Agents Chemother. 2009;53(4):1610–8. doi: 10.1128/AAC.01040-08 19164139 PMC2663061

[52] Wood LF, Ohman DE. Independent regulation of MucD, an HtrA-like protease in Pseudomonas aeruginosa, and the role of its proteolytic motif in alginate gene regulation. J Bacteriol. 2006;188(8):3134–7. doi: 10.1128/JB.188.8.3134-3137.2006 16585775 PMC1447020

[53] Panta PR, Kumar S, Stafford CF, Billiot CE, Douglass MV, Herrera CM. A dedA family membrane protein is required for Burkholderia thailandensis colistin resistance. Frontiers in Microbiology. 2019;10:2532. doi: 10.3389/fmicb.2019.02532 31827463 PMC6849406

[54] Choi K-H, Kumar A, Schweizer HP. A 10-min method for preparation of highly electrocompetent Pseudomonas aeruginosa cells: application for DNA fragment transfer between chromosomes and plasmid transformation. J Microbiol Methods. 2006;64(3):391–7. doi: 10.1016/j.mimet.2005.06.001 15987659

[55] Bradford MM. A rapid and sensitive method for the quantitation of microgram quantities of protein utilizing the principle of protein-dye binding. Anal Biochem. 1976;72. 10.1016/0003-2697(76)90527-3 942051

[56] Livak KJ, Schmittgen TD. Analysis of relative gene expression data using real-time quantitative PCR and the 2−ΔΔCT method. Methods. 2001;25(4):402–8. 11846609 10.1006/meth.2001.1262

