# Supplementary figures and images for: Disruption of undecaprenyl phosphate recycling suppresses ampC beta-lactamase induction in Pseudomonas aeruginosa
Source: PLoS Pathog. 2025 Oct 21;21(10):e1013633. doi: 10.1371/journal.ppat.1013633 (PMC12561984; doi:10.1371/journal.ppat.1013633)

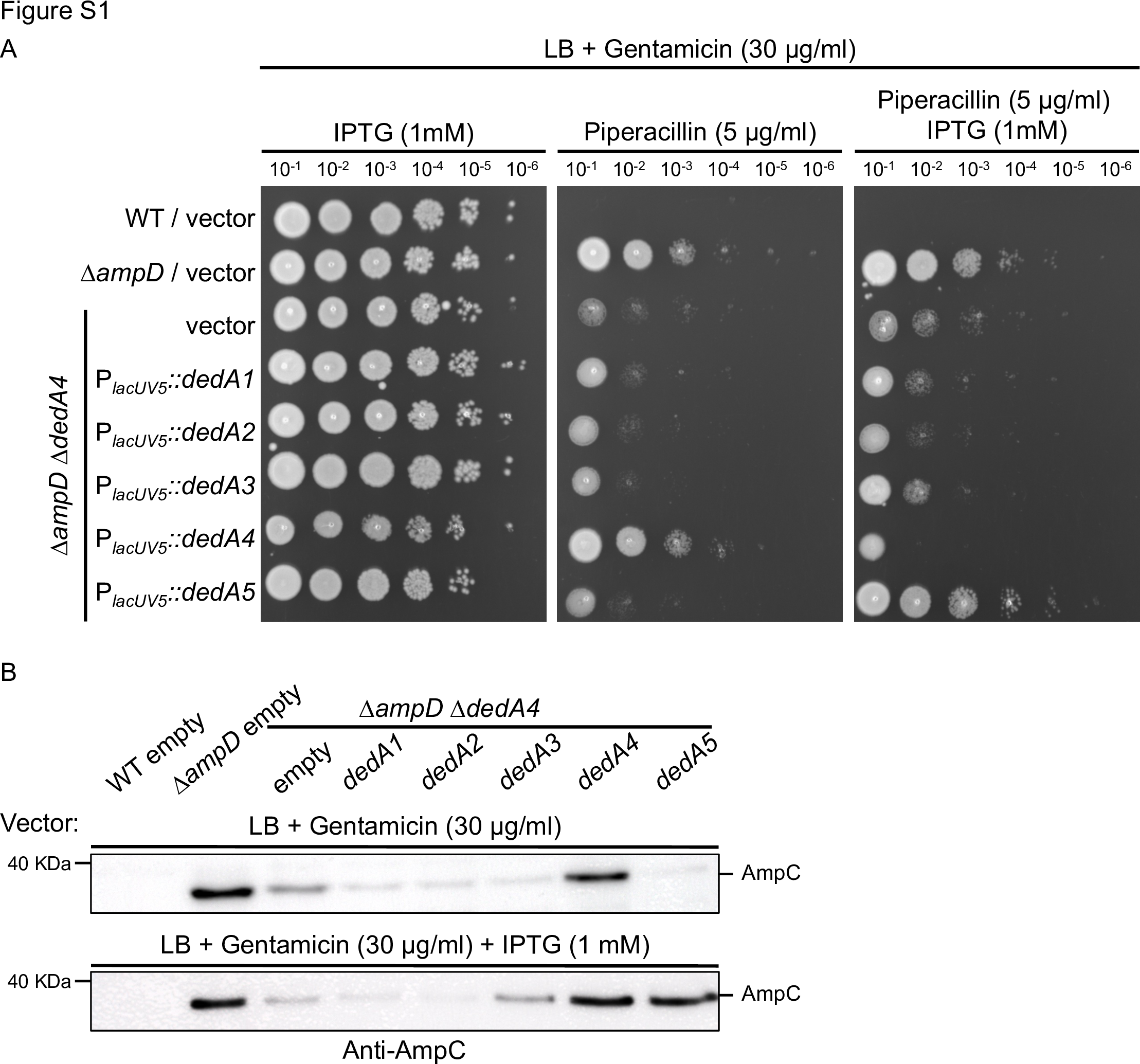

Supplement: S1 Fig — (A) Cultures of the strains PAO1 [WT], CF5 [∆ampD] and CF1844 [∆ampD ∆dedA4], harboring an empty vector (pSV38) or plasmids pCF1141 (PlacUV5::dedA1), pCF1145 (PlacUV5::dedA2), pCF1137 (PlacUV5::dedA3), pCF835 (PlacUV5::dedA4) or pCF577 (PlacUV5::dedA5) were diluted and 5 μl of each dilution was spotted onto LB agar supplemented with gentamicin 30 μg/ml for plasmid maintenance,with or without IPTG inducer and/or piperacillin (5 μg/ml). (B) Immunoblot for AmpC protein using the strains from panel (A) grown in LB supplemented with gentamicin (30 μg/ml) and with or without IPTG (1 mM), as indicated. (TIF) [file ppat.1013633.s001.tif]

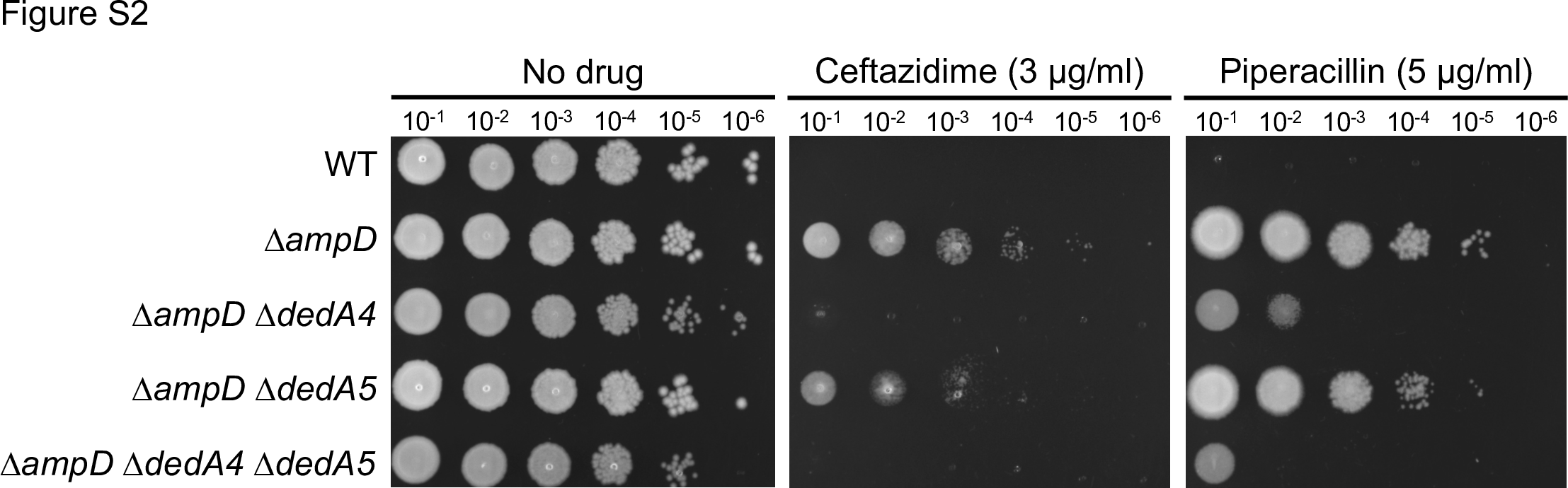

Supplement: S2 Fig — Cultures of the strains PAO1 [WT], CF5 [∆ampD], CF1844 [∆ampD ∆dedA4], CF2041 [∆ampD ∆dedA5] and CF2043 [∆ampD ∆dedA4 ∆dedA5] were diluted and 5 μl of each dilution was spotted onto LB agar supplemented with either ceftazidime (3 μg/ml) or piperacillin (5 μg/ml). (TIF) [file ppat.1013633.s002.tif]

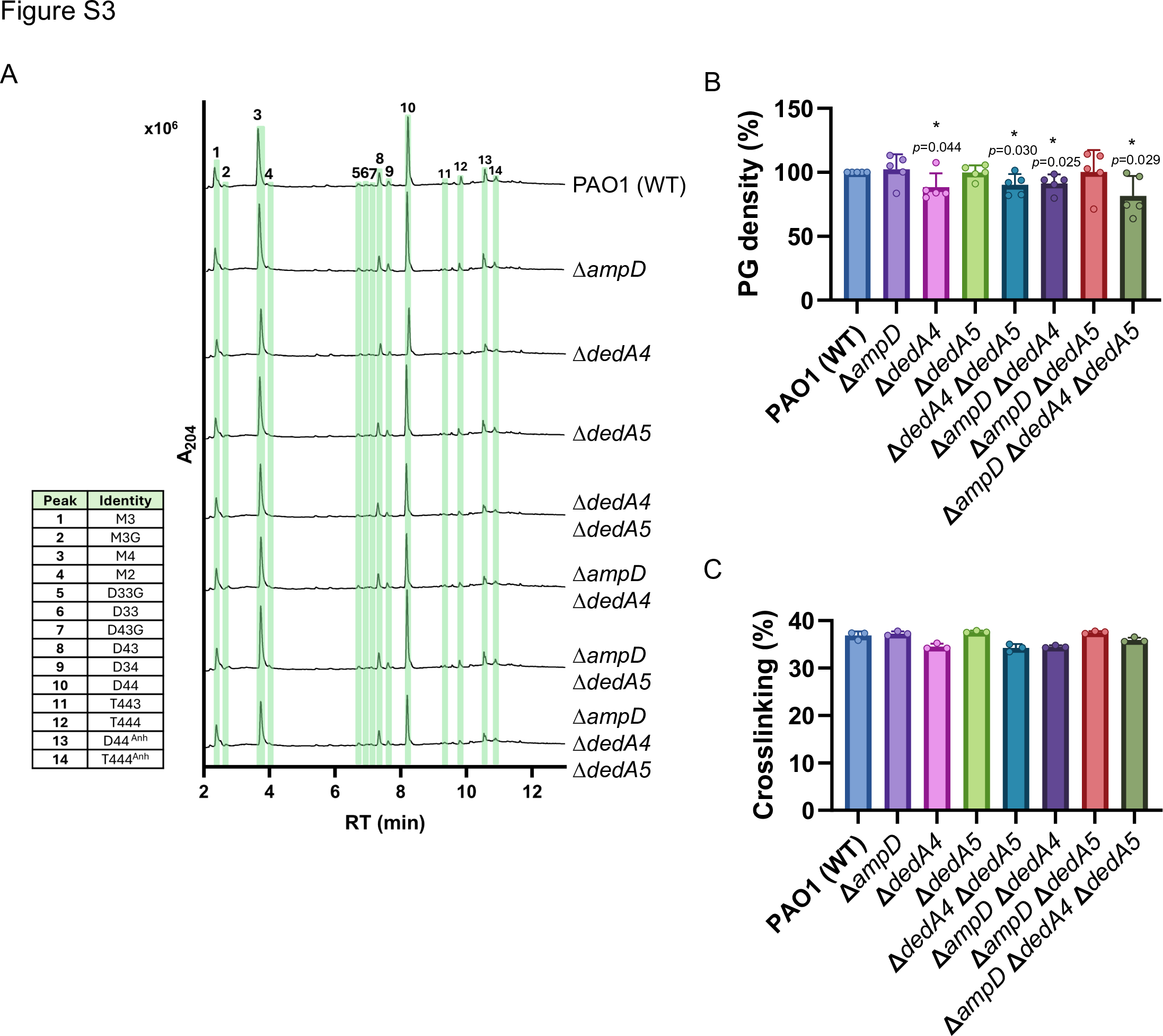

Supplement: S3 Fig — (A) Ultra Performance Liquid Chromatography (UPLC) PG analysis, with characteristic peaks labeled as follows: M = monomeric muropeptide (uncrosslinked), D = dimeric muropeptide (crosslink connecting two muropeptides), T = trimeric muropeptide (crosslinks connecting three muropeptides). Numbers indicate the status of the peptide side chain (3 = tripeptide, 4 = tetrapeptide). Strains used for analysis were: PAO1 [WT], CF5 [∆ampD], CF1842 [∆dedA4], CF2034 [∆dedA5], CF2037 [∆dedA4 ∆dedA5], CF1844 [∆ampD ∆dedA4], CF2041 [∆ampD ∆dedA5] and CF2043 [∆ampD ∆dedA4 ∆dedA5]. (B) Total peptidoglycan content in the same strains as in panel (A). (C) Analysis of PG crosslinking in the same strains as in panel (A). (TIF) [file ppat.1013633.s003.tif]

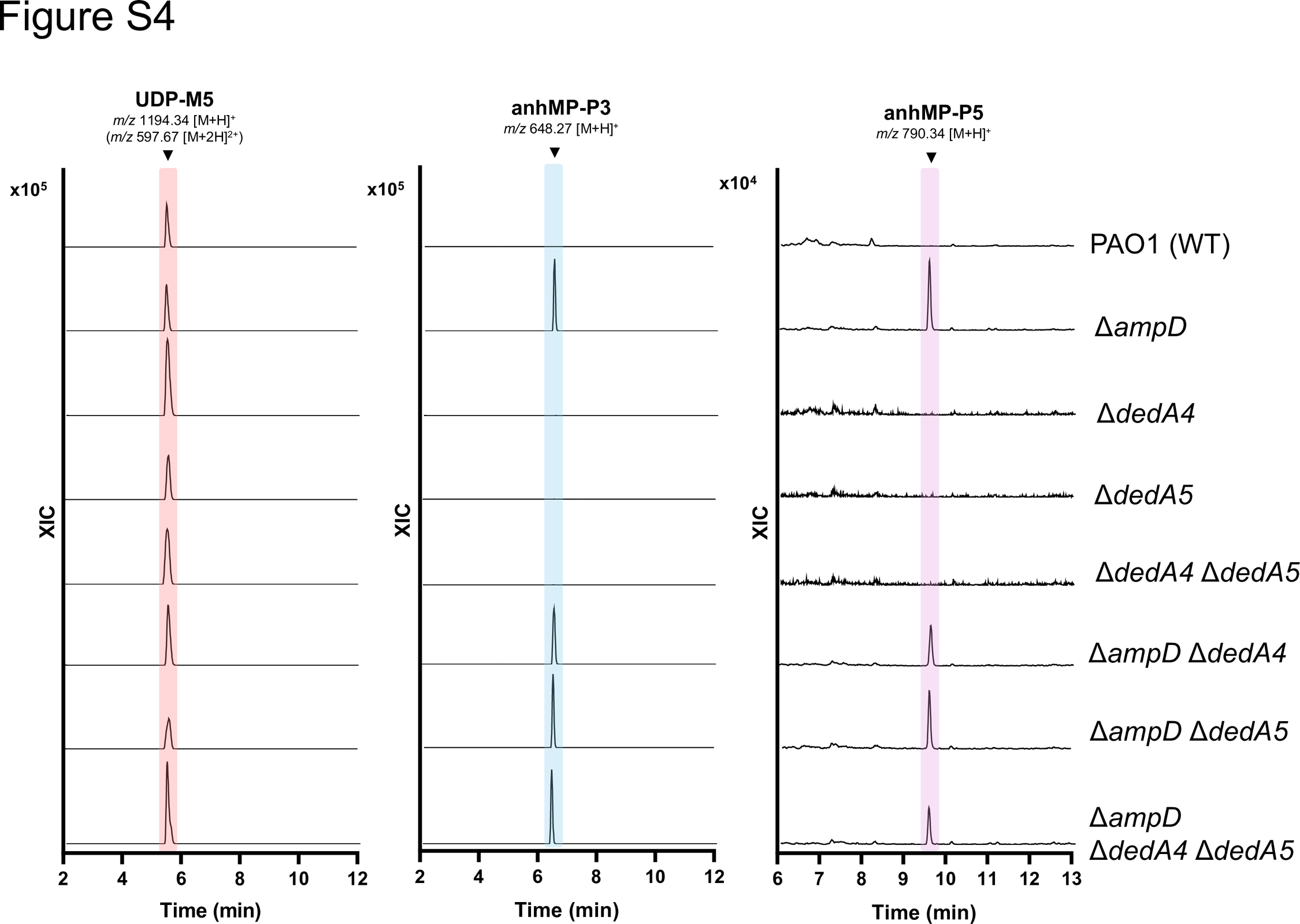

Supplement: S4 Fig — (TIF) [file ppat.1013633.s004.tif]
