# Supplementary material for: Disruption of undecaprenyl phosphate recycling suppresses ampC beta-lactamase induction in Pseudomonas aeruginosa
Source: PLoS Pathog. 2025 Oct 21;21(10):e1013633. doi: 10.1371/journal.ppat.1013633 (PMC12561984; doi:10.1371/journal.ppat.1013633)
Supplement: S1 Table — (DOCX) [file ppat.1013633.s006.docx]

**Table S1. *Pseudomonas aeruginosa* strains used in this study.**

| **Strain** | **Genotype** | **Source/Reference** |  |
| --- | --- | --- | --- |
| PAO1 | *Wild-type* | (1) |  |
| CF5 | PAO1 ∆*ampD (PA4522)* | (2) |  |
| CF1842 | PAO1 ∆*dedA4 (PA4029)* | This study |  |
| CF2034 | PAO1 ∆*dedA5 (PA5244)* | This study |  |
| CF2037 | PAO1 ∆*dedA4* ∆*dedA5* | This study |  |
| CF1844 | | PAO1 ∆*ampD* ∆*dedA4* | This study |
| CF2041 | PAO1 ∆*ampD* ∆*dedA5* | This study |  |
| CF2043 | PAO1 ∆*ampD* ∆*dedA4* ∆*dedA5* | This study |  |
| CF482 | PAO1 ∆*mucD* | This study |  |
| CF1805 | PAO1 ∆*ampD* ∆*mucD* | This study |  |
| CF1857 | PAO1 (P*_lacUV5_*-empty) | This study |  |
| CF1863 | PAO1 ∆*ampD* (P*_lacUV5_*-empty) | This study |  |
| CF1865 | PAO1 ∆*ampD* (P*_lacUV5_*-*ampD*) | This study |  |
| CF1867 | PAO1 ∆*ampD* (P*_lacUV5_*-*dedA4*) | This study |  |
| CF1873 | PAO1 ∆*ampD* ∆*dedA4* (P*_lacUV5_*-empty) | This study |  |
| CF1875 | PAO1 ∆*ampD* ∆*dedA4* (P*_lacUV5_*-*ampD*) | This study |  |
| CF1877 | PAO1 ∆*ampD* ∆*dedA4* (P*_lacUV5_*-*dedA4*) | This study |  |
| CF1926 | PAO1 ∆*ampD* ∆*dedA4* (P*_lacUV5_*-FLAG-*dedA4*) | This study |  |
| CF1946 | PAO1 ∆*ampD* ∆*dedA4* (P*_lacUV5_*-FLAG-*dedA4 D50A*) | This study |  |
| CF1948 | PAO1 ∆*ampD* ∆*dedA4* (P*_lacUV5_*-FLAG-*dedA4 R149A*) | This study |  |
| CF2010 | PAO1 ∆*ampD* ∆*dedA4* (P*_lacUV5_*-FLAG-*dedA4 D50A R149A*) | This study |  |
| CF2002 | PAO1 ∆*ampD* ∆*dedA4* (P*_lacUV5_*-*dedA1 (PA1209)*) | This study |  |
| CF2004 | PAO1 ∆*ampD* ∆*dedA4* (P*_lacUV5_*-*dedA2 (PA2752)*) | This study |  |
| CF2006 | PAO1 ∆*ampD* ∆*dedA4* (P*_lacUV5_*-*dedA3 (PA4011)*) | This study |  |
| CF1940 | PAO1 ∆*ampD* ∆*dedA4* (P*_lacUV5_*-*dedA5 (PA5244)*) | This study |  |
| CF1998 | PAO1 ∆*ampD* ∆*dedA4* (P*_lacUV5_*-*^Ec^yqjA*) | This study |  |
| CF2000 | PAO1 ∆*ampD* ∆*dedA4* (P*_lacUV5_*- *^Ec^yghB*) | This study |  |
| CF2156 | PAO1 (P_ara_-empty) | This study |  |
| CF2162 | PAO1 ∆*dedA4* (P_ara_-empty) | This study |  |
| CF2164 | PAO1 ∆*dedA4* (P_ara_-*uppS (PA3652)*) | This study |  |
| CF2168 | PAO1 ∆*ampD* (P_ara_-empty) | This study |  |
| CF2172 | PAO1 ∆*ampD* (P_ara_-*murA (PA4450*) | This study |  |

**References**

1. Stover CK, Pham XQ, Erwin AL, Mizoguchi SD, Warrener P, Hickey MJ, et al. Complete genome sequence of Pseudomonas aeruginosa PAO1, an opportunistic pathogen. Nature. 2000 Aug 31;406(6799):959–64.

2. Gyger J, Torrens G, Cava F, Bernhardt TG, Fumeaux C. A potential space-making role in cell wall biogenesis for SltB1and DacB revealed by a beta-lactamase induction phenotype in Pseudomonas aeruginosa. mBio [Internet]. 2024 Jun 26 [cited 2024 Jun 26]; Available from: https://journals.asm.org/doi/10.1128/mbio.01419-24
