# Supplementary material for: Disruption of undecaprenyl phosphate recycling suppresses ampC beta-lactamase induction in Pseudomonas aeruginosa
Source: PLoS Pathog. 2025 Oct 21;21(10):e1013633. doi: 10.1371/journal.ppat.1013633 (PMC12561984; doi:10.1371/journal.ppat.1013633)
Supplement: S2 Table — (DOCX) [file ppat.1013633.s007.docx]

**S2 Table. *Escherichia coli* strains used in this study.**

| **Strain** | **Genotype^a^** | **Source/Reference^b^** |
| --- | --- | --- |
| DH5α | *F– hsdR17 deoR recA1 endA1 phoA supE44 thi-1 gyrA96 relA1 Δ(lacZYA-argF)U169* ϕ*80dlacZΔM15* | Gibco BRL |
| Sm10(λpir) | *Kan^R^ thi-1 thr leu tonA lacY supE recA::RP4-2-Tc::Mu attλ::pir* | (1) |
| MG1655 | *rph-1 ilvG rfb-50* | (2) |

**References**

1. Simon R, Priefer U, Pühler A. A Broad Host Range Mobilization System for In Vivo Genetic Engineering: Transposon Mutagenesis in Gram Negative Bacteria. Bio/Technology. 1983 Nov 1;1(9):784–91.

2. Guyer MS, Reed RR, Steitz JA, Low KB. Identification of a sex-factor-affinity site in E. coli as gamma delta. Cold Spring Harb Symp Quant Biol. 1981;45 Pt 1:135–40.
