# Supplementary material for: Disruption of undecaprenyl phosphate recycling suppresses ampC beta-lactamase induction in Pseudomonas aeruginosa
Source: PLoS Pathog. 2025 Oct 21;21(10):e1013633. doi: 10.1371/journal.ppat.1013633 (PMC12561984; doi:10.1371/journal.ppat.1013633)
Supplement: S3 Table — (DOCX) [file ppat.1013633.s008.docx]

**Table S3.** Plasmids used in this study.

| ***Plasmid*** | ***Genotype*** | ***ori*** | ***Source/Reference*** |
| --- | --- | --- | --- |
| pEXG2 | *aacC1 sacB oriT [vector for allelic exchange in P. aeruginosa]* | pBR/colE1 | (1) |
| pJN105 | *aacC1 araC* P_ara_ *[replicating arabinose-inducible expression vector for P. aeruginosa]* | pBBR1 | (2) |
| pPSV38 | *aacC1 lacIq* P*_lacUV5_* [replicating IPTG-inducible expression vector for *P. aeruginosa*] | pBR322/ pRO1600 | (3) |
| pCF579 | *aacC1 sacB oriT ‘PA4522-ampD∆ (2-187)’* | pBR/colE1 | (4) |
| pCF833 | *aacC1 sacB oriT ‘PA4029-dedA4∆ (10-216)’* | pBR/colE1 | This study |
| pCF1001 | *aacC1 sacB oriT ‘PA5244-dedA5∆ (10-189)’* | pBR/colE1 | This study |
| pCF219 | *aacC1 sacB oriT ‘PA0766-mucD∆ (14-464)’* | pBR/colE1 | This study |
| pCF1098 | *aacC1 lacIq* P*_lacUV5_-ampD* | pBR322/ pRO1600 | This study |
| pCF1141 | *aacC1 lacIq* P*_lacUV5_-dedA1* | pBR322/ pRO1600 | This study |
| pCF1145 | *aacC1 lacIq* P*_lacUV5_-dedA2* | pBR322/ pRO1600 | This study |
| pCF1137 | *aacC1 lacIq* P*_lacUV5_-dedA3* | pBR322/ pRO1600 | This study |
| pCF835 | *aacC1 lacIq* P*_lacUV5_-dedA4* | pBR322/ pRO1600 | This study |
| pCF577 | *aacC1 lacIq* P*_lacUV5_-dedA5* | pBR322/ pRO1600 | This study |
| pCF661 | *aacC1 lacIq* P*_lacUV5_-*FLAG*-dedA4* | pBR322/ pRO1600 | This study |
| pCF580 | *aacC1 lacIq* P*_lacUV5_-*FLAG*-dedA4 D50A* | pBR322/ pRO1600 | This study |
| pCF584 | *aacC1 lacIq* P*_lacUV5_-*FLAG*-dedA4 R149A* | pBR322/ pRO1600 | This study |
| pCF1154 | *aacC1 lacIq* P*_lacUV5_-*FLAG*-dedA4 D50A R149A* | pBR322/ pRO1600 | This study |
| pCF1147 | *aacC1 lacIq* P*_lacUV5_-^Ec^yqjA* | pBR322/ pRO1600 | This study |
| pCF1150 | *aacC1 lacIq* P*_lacUV5_-^Ec^yghB* | pBR322/ pRO1600 | This study |
| pCF214 | *aacC1 araC* P_ara_:: RBS_optimized_-*uppS (PA3652)* | pBBR1 | This study |
| pCF166 | *aacC1 araC* P_ara_:: RBS_optimized_-*murA (PA4450)* | pBBR1 | This study |

**References**

1. Rietsch A, Vallet-Gely I, Dove SL, Mekalanos JJ. ExsE, a secreted regulator of type III secretion genes in Pseudomonas aeruginosa. Proc Natl Acad Sci U S A. 2005 May 31;102(22):8006–11.

2. Newman JR, Fuqua C. Broad-host-range expression vectors that carry the L-arabinose-inducible Escherichia coli araBAD promoter and the araC regulator. Gene. 1999 Feb 18;227(2):197–203.

3. Vvedenskaya IO, Sharp JS, Goldman SR, Kanabar PN, Livny J, Dove SL, et al. Growth phase-dependent control of transcription start site selection and gene expression by nanoRNAs. Genes Dev. 2012 Jan 7;26(13):1498–507.

4. Gyger J, Torrens G, Cava F, Bernhardt TG, Fumeaux C. A potential space-making role in cell wall biogenesis for SltB1and DacB revealed by a beta-lactamase induction phenotype in Pseudomonas aeruginosa. mBio [Internet]. 2024 June 26 [cited 2024 June 26]; Available from: https://journals.asm.org/doi/10.1128/mbio.01419-24
