# Supplementary material for: Disruption of undecaprenyl phosphate recycling suppresses ampC beta-lactamase induction in Pseudomonas aeruginosa
Source: PLoS Pathog. 2025 Oct 21;21(10):e1013633. doi: 10.1371/journal.ppat.1013633 (PMC12561984; doi:10.1371/journal.ppat.1013633)
Supplement: S4 Table — (DOCX) [file ppat.1013633.s009.docx]

**S4 Table.** Identified muropeptides.

| **Identity** | **Proposed Structure** | **Observed *m/z* value** | **Observations** |
| --- | --- | --- | --- |
| M3 | GlcNAc-MurNAc-L-Ala-D-Glu-m-DAP | 871.3784 | [M+H]+ |
| M3G | GlcNAc-MurNAc-L-Ala-D-Glu-m-DAP-Gly | 928.3992 | [M+H]+ |
| M4 | GlcNAc-MurNAc-L-Ala-D-Glu-m-DAP-D-Ala | 942.4156 | [M+H]+ |
| M2 | GlcNAc-MurNAc-L-Ala-D-Glu | 699.2936 | [M+H]+ |
| D33G | M3-M3G (DAP-m-DAP crosslink) | 890.8839 | [M+2H]2+ |
| D33 | M3-M3 (DAP-m-DAP crosslink) | 862.3731 | [M+2H]2+ |
| D43G | M4-M3G (D-Ala-m-DAP crosslinks) | 926.4024 | [M+2H]2+ |
| D43 | M4-M3 (D-Ala-m-DAP crosslink) | 897.8917 | [M+2H]2+ |
| D34 | M3-M4 (DAP-m-DAP crosslink) | 897.8917 | [M+2H]2+ |
| D44 | M4-M4 (D-Ala-m-DAP crosslink) | 933.4102 | [M+2H]2+ |
| T443 | M4-M4-M3 (D-Ala-m-DAP crosslink) | 906.7295 | [M+3H]3+ |
| T444 | M4-M4-M4 (D-Ala-m-DAP crosslink) | 930.4085 | [M+3H]3+ |
| D44 ^Anh^ | M4-M4^Anh^ (D-Ala-m-DAP crosslink) | 923.3971 | [M+2H]2+ |
| T444^Anh^ | M4-M4-M4^Anh^ (D-Ala-m-DAP crosslink) | 923.7331 | [M+3H]3+ |
| UDP-M5 | UDP-MurNAc-L-Ala-D-Glu-m-DAP-D-Ala-D-Ala | 1194.3492 | [M+H]+ |
| NAM^Anh^-P3 | Anhydro-MurNAc-L-Ala-D-Glu-m-DAP | 648.2728 | [M+H]+ |
| NAM^Anh^-P5 | Anhydro-MurNAc-L-Ala-D-Glu-m-DAP-D-Ala-D-Ala | 790.3470 | [M+H]+ |

*GlcNAc: N-acetylglucosamine; MurNAc: N-acetylmuramic acid (NAM); Anh: anhydro N-acetylmuramic acid; L/D-Ala: alanine; D-Glu: glutamic acid; m-DAP: meso-diaminopimelic acid.
