# Supplementary material for: Disruption of undecaprenyl phosphate recycling suppresses ampC beta-lactamase induction in Pseudomonas aeruginosa
Source: PLoS Pathog. 2025 Oct 21;21(10):e1013633. doi: 10.1371/journal.ppat.1013633 (PMC12561984; doi:10.1371/journal.ppat.1013633)
Supplement: S5 Table — (DOCX) [file ppat.1013633.s010.docx]

**S5 Table.** Primers used for RT-qPCR.

| **Target** | **Sequence** |
| --- | --- |
| *rpsL* | 5’-GCT GCA AAA CTG CCC GCA ACG-3’ |
|  | 5’-ACC CGA GGT GTC CAG CGA ACC-3’ |
| *gyrB* | 5’-CCT GAC CAT CCG TCG CCA CAA C-3’ |
|  | 5’-ACA GCT CCT CCT TGC CGG TAC G-3’ |
| *ampC* | 5’-GGG CTG GCC TCG AAA GAG GAC-3’ |
|  | 5’-GCA CCG AGT CGG GGA ACT GCA-3’ |
