# Supplementary material for: Disruption of undecaprenyl phosphate recycling suppresses ampC beta-lactamase induction in Pseudomonas aeruginosa
Source: PLoS Pathog. 2025 Oct 21;21(10):e1013633. doi: 10.1371/journal.ppat.1013633 (PMC12561984; doi:10.1371/journal.ppat.1013633)
Supplement: S1 Appendix — (DOCX) [file ppat.1013633.s011.docx]

**S1 Appendix**

**Plasmid construction:**

For all plasmid constructions (**Table S3**), PCR was performed using Phusion (F530L, Thermo Fisher Scientific) or Q5 DNA polymerase (M0491S, New England Biolabs) according to the manufacturer’s instructions. Unless otherwise indicated, PAO1 chromosomal DNA was used as the template. Restriction sites for use in plasmid constructions are italicized and underlined in the primer sequences given below. Plasmid DNA and PCR fragments were purified using the GeneJET miniprep kit (Thermo Fisher Scientific) and the NucleoSpin Gel and PCR clean up (Macherey-Nagel AG), respectively. All plasmids were verified using the Microsynth AG company Sanger sequencing service.

To construct **pCF833** [*aacC1 sacB oriT ‘PA4029-dedA4∆ (10-216)’*], which is used for deletion of *dedA4* (*PA4029*), the ~600bp region upstream of *dedA4* was amplified from PAO1 genomic DNA (gDNA) using 5’-AAA *AAG CTT* AAA GTG CAC GCC TCC GGC ATG GAC CA-3’ and 5’-ACC ACA TGC TCA GTC GCT CTG GGC CTT AAT CAG ATC GAT TGG GTT GAA GTC CAT-3’ primers. The ~850bp region downstream of the gene was amplified with 5’-ATG GAC TTC AAC CCA ATC GAT CTG ATT AAG GCC CAG AGC GAC TGA GCA TGT GGT-3’ and 5’-AAA *GGA TCC* ATT CAG CCG TTA GTC GTG TCG CC-3’ primers. The two resulting fragments were then combined by sewing PCR with 5’-AAA *AAG CTT* AAA GTG CAC GCC TCC GGC ATG GAC CA-3’ and 5’-AAA *GGA TCC* ATT CAG CCG TTA GTC GTG TCG CC-3’ primers. This PCR fragment was then digested with HindIII and BamHI and ligated with appropriately digested pEXG2 (1) to generate pCF833.

To construct **pCF1001** [*aacC1 sacB oriT ‘PA5244-dedA5∆ (10-189)’*], which is used for deletion of *dedA5* (*PA5244*), the ~600bp region upstream of *dedA5* was amplified from PAO1 genomic DNA (gDNA) using 5’-AAA *GAA TTC* AAA GCG CTC GCG CAG GGC ACG GGT-3’ and 5’-TTC ACT CGG ATT TGT CGG CGT TGC CGT CCT GCA GGA ATT GTT GGA GCA TCA T-3’ primers. The ~600bp region downstream of the gene was amplified with 5’-ATG ATG CTC CAA CAA TTC CTG CAG GAC GGC AAC GCC GAC AAA TCC GAG TGA A-3’ and 5’-AAA *TCT AGA* AAG CCT TGG CCA GCG CCA GCA CGT-3’ primers. The two resulting fragments were then combined by sewing PCR with 5’-AAA *GAA TTC* AAA GCG CTC GCG CAG GGC ACG GGT-3’ and 5’-AAA *TCT AGA* AAG CCT TGG CCA GCG CCA GCA CGT-3’ primers. This PCR fragment was then digested with EcoRI and XbaI and ligated with appropriately digested pEXG2 (1) to generate pCF1001.

To construct **pCF219** [*aacC1 sacB oriT ‘PA0766-mucD∆ (14-464)’*], which is used for deletion of *mucD* (*PA0766*), the ~600bp region upstream of *mucD* was amplified from PAO1 genomic DNA (gDNA) using 5’-AAA *AAG CTT* CCA GAT GGT GAC CGT CGT CGG CGA AGT-3’ and 5’-GGC CAG CTT GAA GGT AAT GAA GCT GGC CAC CAT CGC AGC CAT ACA GCG-3’ primers. The ~600bp region downstream of the gene was amplified with 5’-CGC TGT ATG GCT GCG ATG GTG GCC AGC TTC ATT ACC TTC AAG CTG GCC-3’ and 5’-AAA *GGT ACC* GAT GAT GCT CTC GAT TTC CTC TTT-3’ primers. The two resulting fragments were then combined by sewing PCR with 5’-AAA *AAG CTT* CCA GAT GGT GAC CGT CGT CGG CGA AGT-3’ and 5’-AAA *GGT ACC* GAT GAT GCT CTC GAT TTC CTC TTT-3’ primers. This PCR fragment was then digested with HindIII and KpnI and ligated with appropriately digested pEXG2 (1) to generate pCF219.

For **pCF1098** [*aacC1 lacIq* P*_lacUV5_*:: RBS_optimized_-*ampD*], which is used for expression of *ampD* (*PA4522*), the gene was amplified from PAO1 genomic DNA (gDNA) using 5’-AAA *GAG CTC* **GAG GAG GAT ACA T**AT GCA TTT CGA TTC CGT TAC CGG CT-3’ and 5’-AAA *TCT AGA* TCA TGT TTC CTC CTT GGA ATC GGT C-3’ primers. The synthetic RBS is bolded. The PCR product was digested with SacI and XbaI and ligated into similarly digested pPSV38 (2) to generate pCF1098.

For **pCF1141** [*aacC1 lacIq* P*_lacUV5_*:: RBS_optimized_-*dedA1*], which is used for expression of *dedA1* (*PA1209*), the gene was amplified from PAO1 genomic DNA (gDNA) using 5’-AAA *GAG CTC* **GAG GAG GAT ACA T** ATG CGT GAA CTG CAA CCC CTG CTG-3’ and 5’-AAA *TCT AGA* TCA GGC GTT CGC CAC GAC CTG CC-3’ primers. The synthetic RBS is bolded. The PCR product was digested with SacI and XbaI and ligated into similarly digested pPSV38 (2) to generate pCF1141.

For **pCF1145** [*aacC1 lacIq* P*_lacUV5_*:: RBS_optimized_-*dedA2*], which is used for expression of *dedA2* (*PA2752*), the gene was amplified from PAO1 genomic DNA (gDNA) using 5’-AAA *GAG CTC* **GAG GAG GAT ACA T** GTG TTG ACT GAC TGG GCC GCG TAC-3’ and 5’-AAA *TCT AGA* TCA GCC GGC CCA GCC CAG CAC CA-3’ primers. The synthetic RBS is bolded. The PCR product was digested with SacI and XbaI and ligated into similarly digested pPSV38 (2) to generate pCF1145.

For **pCF1137** [*aacC1 lacIq* P*_lacUV5_*:: RBS_optimized_-*dedA3*], which is used for expression of *dedA3* (*PA4011*), the gene was amplified from PAO1 genomic DNA (gDNA) using 5’-AAA *GAG CTC* **GAG GAG GAT ACA T**AT GAG TCT CGA CAG CTT CAA CGC CT-3’ and 5’-AAA *TCT AGA* TCA CAG CGG CCG GTA GCG CGC AAT-3’ primers. The synthetic RBS is bolded. The PCR product was digested with SacI and XbaI and ligated into similarly digested pPSV38 (2) to generate pCF1137.

For **pCF835** [*aacC1 lacIq* P*_lacUV5_*:: RBS_optimized_-*dedA4*], which is used for expression of *dedA4* (*PA4029*), the gene was amplified from PAO1 genomic DNA (gDNA) using 5’-AAA *GAG CTC* **GAG GAG GAT ACA T** AT GGA CTT CAA CCC AAT CGA TCT G-3’ and 5’-AAA *TCT AGA* TCA GTC GCT CTG GGC CTT GGC CTG TT-3’ primers. The synthetic RBS is bolded. The PCR product was digested with SacI and XbaI and ligated into similarly digested pPSV38 (2) to generate pCF835.

For **pCF577** [*aacC1 lacIq* P*_lacUV5_*:: RBS_optimized_-*dedA5*], which is used for expression of *dedA5* (*PA5244*), the gene was amplified from PAO1 genomic DNA (gDNA) using 5’-AAA *GAG CTC* **GAG GAG GAT ACA T**AT GAT GCT CCA ACA ATT CCT GCA G-3’ and 5’-AAA *TCT AGA* TCA CTC GGA TTT GTC GGC GTT GCC-3’ primers. The synthetic RBS is bolded. The PCR product was digested with SacI and XbaI and ligated into similarly digested pPSV38 (2) to generate pCF577.

For **pCF661** [*aacC1 lacIq* P*_lacUV5_*:: RBS_optimized_-FLAG-*dedA4*], which is used for expression of a N-terminal flagged *dedA4* (*PA4029*), the gene was amplified from PAO1 genomic DNA (gDNA) using 5’-AAA *GAG CTC* **GAG GAG GAT ACA T**AT G**GA CTA TAA GGA TGA TGA TGA TAA G**GA CTT CAA CCC AAT CGA TCT G-3’ and 5’-AAA *TCT AGA* TCA GTC GCT CTG GGC CTT GGC CTG TT-3’ primers. The synthetic RBS and the FLAG tag are bolded. The PCR product was digested with SacI and XbaI and ligated into similarly digested pPSV38 (2) to generate pCF661.

For **pCF580** [*aacC1 lacIq* P*_lacUV5_*:: RBS_optimized_-FLAG-*dedA4 D50A*], which is used for expression of a N-terminal flagged *dedA4 D50A* (*PA4029*) catalytic mutant, was created in several steps. First, the 5’ end of *dedA4* gene was PCR-amplified from PAO1 gDNA with 5’-AAA *GAG CTC* **GAG GAG GAT ACA T**AT G**GA CTA TAA GGA TGA TGA TGA TAA G**GA CTT CAA CCC AAT CGA TCT G-3’ and *dedA4 D50A* #2 (5’-ATG AAC AGC AGC GAG GCG CCG GGG AGG AA-3’) primers. Concurrently, the 3’ end of *dedA4* gene was PCR-amplified from PAO1 gDNA with *dedA4 D50A* #1 (5’-TTC CTC CCC GGC GCC TCG CTG CTG TTC AT-3’) and 5’-AAA *TCT AGA* TCA GTC GCT CTG GGC CTT GGC CTG TT-3’ primers. The synthetic RBS and the FLAG tag are bolded. The mutated site is underlined. These two PCR products were then combined by sewing PCR with 5’-AAA *GAG CTC* **GAG GAG GAT ACA T**AT G**GA CTA TAA GGA TGA TGA TGA TAA G**GA CTT CAA CCC AAT CGA TCT G-3 and 5’-AAA *TCT AGA* TCA GTC GCT CTG GGC CTT GGC CTG TT-3’ primers. This final PCR product was digested with SacI and XbaI and ligated into similarly digested pPSV38 (2) to generate pCF580.

**pCF584** [*aacC1 lacIq* P*_lacUV5_*:: RBS_optimized_-FLAG-*dedA4 R149A*], which is used for expression of a N-terminal flagged *dedA4 R149A* (*PA4029*) catalytic mutant, was created as described above for pCF580, using primers *dedA4 R149A* #2 (5’-ACG ATG GGC AGG AAG GCC GCC AGG GTC ACG GT-3’) and *dedA4 R149A* #1 (5’- ACC GTG ACC CTG GCG GCC TTC CTG CCC ATC GT-3’).

**pCF1154** [*aacC1 lacIq* P*_lacUV5_*:: RBS_optimized_-FLAG-*dedA4 D50A R149A*], which is used for expression of a N-terminal flagged *dedA4 D50A R149A* (*PA4029*) double mutant, was created as described above for pCF584, using primers *dedA4 R149A* #2 (5’-ACG ATG GGC AGG AAG GCC GCC AGG GTC ACG GT-3’) and *dedA4 R149A* #1 (5’- ACC GTG ACC CTG GCG GCC TTC CTG CCC ATC GT-3’), but pCF580 was used as a template for the initial PCRs instead of PAO1 gDNA.

For **pCF1147** [*aacC1 lacIq* P*_lacUV5_*:: RBS_optimized_-*^Ec^yqjA*], which is used for expression of *E. coli yqjA*, the gene was amplified from *Escherichia coli* MG1655 genomic DNA (gDNA) using 5’-AAA *GAG CTC* **GAG GAG GAT ACA T**AT GGA ACT TTT GAC CCA ATT GCT G-3’ and 5’-AAA *TCT AGA* TTA CCC CCG ATT TCC ATA TTT CT-3’ primers. The synthetic RBS is bolded. The PCR product was digested with SacI and XbaI and ligated into similarly digested pPSV38 (2) to generate pCF1147.

For **pCF1150** [*aacC1 lacIq* P*_lacUV5_*:: RBS_optimized_-*^Ec^yghB*], which is used for expression of *E. coli yghB*, the gene was amplified from *Escherichia coli* MG1655 genomic DNA (gDNA) using 5’-AAA *GAG CTC* **GAG GAG GAT ACA T**AT GGC TGT TAT TCA AGA TAT CAT C-3’ and 5’-AAA *TCT AGA* TCA GGC GTT ACA GTA TTT TTT TT-3’ primers. The synthetic RBS is bolded. The PCR product was digested with SacI and XbaI and ligated into similarly digested pPSV38 (2) to generate pCF1150.

For **pCF166** [*aacC1 araC* P_ara_:: RBS_optimized_-*murA*], which is used for expression of *murA* (*PA4450*), the gene was amplified from PAO1 genomic DNA (gDNA) using 5’-AAA *GAA TTC* **GAG GAG GA**T TGC AAT GGA TAA ACT GAT TAT T-3’ and 5’- AAA *TCT AGA* CTT GGA CAA GGC GAT GGT CAG CAT-3’ primers. The optimized RBS is bolded. The PCR product was digested with EcoRI and XbaI and ligated into similarly digested pJN105 (3) to generate pCF166.

**pCF214** [*aacC1 araC* P_ara_:: RBS_optimized_-*uppS*], which is used for expression of *uppS* (*PA3652*), the gene was amplified from PAO1 genomic DNA (gDNA) using 5’-AAA *GAA TTC* **GAG GAG GAT C**GT CAT GGA AAA GAC CCG GAA GGA-3’ and 5’-AAA *TCT AGA* CGT GAT GAT CCG TTG TTT CAG CAT-3’ primers. The optimized RBS is bolded. The PCR product was digested with EcoRI and XbaI and ligated into similarly digested pJN105 (3) to generate pCF214.

***P. aeruginosa* strain construction.**

During construction *of P. aeruginosa* deletion strains, plasmids were transferred into *P. aeruginosa* by conjugation from an *E. coli* donor [SM10(λpir)] on LB plates. Counter-selection against *E. coli* was accomplished on Vogel-Bonner minimal medium (VBMM)(4) supplemented with 30 μg/ml gentamicin.

To create the Δ*dedA4* strains CF1842 [PAO1∆*dedA4*] and CF1844 [PAO1∆*ampD*∆*dedA4*], pCF833 [*aacC1 sacB oriT ‘PA4029-dedA4∆ (10-216)’*] was conjugated into PAO1 [WT] and CF5 [PAO1 ∆*ampD*] recipient from SM10(λpir) donor. For this purpose, PAO1 and CF5 were patched on an LB plate and grown overnight at 42°C while SM10(λpir) carrying pCF833 was similarly grown at 37°C. Both the donor and the recipients were scraped, patched together onto an LB plate, and incubated at 37°C for ~5h. The cells were scraped, resuspended in 500 μL of VBMM, diluted 1:10, and 100 μL of the resulting suspension was plated on VBMM supplemented with 30 μg/mL gentamicin. Plates were incubated at 37°C overnight. The exconjugants were purified on LB supplemented with 30 μg/mL gentamicin. A few single colonies were allowed to grow for ~6h in plain LB broth to allow for the second plasmid recombination event, and 100 μL of the resulting culture was plated on LB supplemented with 5% (w/v) sucrose to select for the loss of the plasmid-encoded *sacB* gene. Sucrose-resistant colonies were then patched onto LB plates either containing or lacking 30 μg/mL gentamicin. Gentamicin-sensitive colonies were further tested by PCR with *dedA4*-flanking primers 5’-AAC ATC CTG CAA TCA GTG GTT T-3’ and 5’-AAG CCC TGG TAC CAG TTC AGC TCC-3’ to confirm gene deletion. The deletion retains the first nine and last six codons of the *dedA4* reading frame.

To create the Δ*dedA5* strains CF2034 [PAO1∆*dedA5*], CF2037 [PAO1∆*dedA4*∆*dedA5*], CF2041 [PAO1∆*ampD*∆*dedA5*] and CF2043 [PAO1∆*ampD*∆*dedA4*∆*dedA5*], *dedA5* was deleted from PAO1 [WT], CF1842 [PAO1∆*dedA4*], CF5 [PAO1∆*ampD*] and CF1844 [PAO1∆*ampD*∆*dedA4*] by integration and re-circularization of pCF1001 [*aacC1 sacB oriT ‘PA5244-dedA5∆ (10-189)’*] as described above. Sucrose-resistant, gentamicin-sensitive colonies were screened by PCR with *dedA5*-flanking primers 5’-AAC GGG TCG AGC GCC ACG TCG GT-3’ and 5’-AGA TCA GCC CGA CTG GCT TGC-3’. The deletion retains the first nine and last eight codons of the *dedA5* reading frame.

To create the Δ*mucD* strains CF482 [PAO1∆*mucD*] and CF1805 [PAO1∆*ampD* ∆*mucD*], *mucD* was deleted from PAO1 [WT] and CF5 [PAO1∆*ampD*] by integration and re-circularization of pCF219 [*aacC1 sacB oriT ‘PA0766-mucD∆ (14-464)’*] as described above. Sucrose-resistant, gentamicin-sensitive colonies were screened by PCR with *mucD*-flanking primers 5’- AAC CTC TGA TCA TCG TGT CCG GG-3’ and 5’- AAG CGA TCG GCC AGC GTC GAC TT-3’. The deletion retains the first thirteen and last ten codons of the *mucD* reading frame.

During construction *of P. aeruginosa* expression strains, plasmids were transferred into *P. aeruginosa* by electroporation using the following settings: 25 mF, 200 O, 2.5 kV (Gene Pulser, Bio-Rad).

Strain CF1857 [PAO1 (P*_lacUV5_*-empty)], was obtained by electroporation of plasmid pPSV38 (2), in PAO1 [WT].

Strains CF1863 [∆*ampD* (P*_lacUV5_*-empty)], CF1865 [∆*ampD* (P*_lacUV5_*:: RBS_optimized_-*ampD*)] and CF1867 [∆*ampD* (P*_lacUV5_*:: RBS_optimized_-*dedA4*)] were obtained by electroporation of plasmids pPSV38 (2), pCF1098 and pCF835 in CF5 [∆*ampD*] (5), respectively.

Strains CF1873 [∆*ampD*∆*dedA4* (P*_lacUV5_*-empty)], CF1875 [∆*ampD*∆*dedA4* (P*_lacUV5_*:: RBS_optimized_-*ampD*)] CF2002 [∆*ampD*∆*dedA4* (P*_lacUV5_*:: RBS_optimized_-*dedA1*)], CF2004 [∆*ampD*∆*dedA4* (P*_lacUV5_*:: RBS_optimized_-*dedA2*)], CF2006 [∆*ampD*∆*dedA4* (P*_lacUV5_*:: RBS_optimized_-*dedA3*)], CF1877 [∆*ampD*∆*dedA4* (P*_lacUV5_*:: RBS_optimized_-*dedA4*)], CF1940 [∆*ampD*∆*dedA4* (P*_lacUV5_*:: RBS_optimized_-*dedA5*)], CF1926 [∆*ampD*∆*dedA4* (P*_lacUV5_*:: RBS_optimized_-FLAG-*dedA4*)], CF1946 [∆*ampD*∆*dedA4* (P*_lacUV5_*:: RBS_optimized_-FLAG-*dedA4 D50A*)], CF1948 [∆*ampD*∆*dedA4* (P*_lacUV5_*:: RBS_optimized_-FLAG-*dedA4 R149A*)], CF2010 [∆*ampD*∆*dedA4* (P*_lacUV5_*:: RBS_optimized_-FLAG-*dedA4 D50A R149A*)], CF1998 [∆*ampD*∆*dedA4* (P*_lacUV5_*:: RBS_optimized_-*^Ec^yqjA*)] and CF2000 [∆*ampD*∆*dedA4* (P*_lacUV5_*:: RBS_optimized_-*^Ec^yghB*)] were obtained by electroporation of plasmids pPSV38 (2), pCF1098, pCF1141, pCF1145, pCF1137, pCF835, pCF577, pCF661, pCF580, pCF584, pCF1154, pCF1147 and pCF1150 in CF1844 [∆*ampD*∆*dedA4*], respectively.

Strain CF2156 [PAO1 (P_ara_-empty)], was obtained by electroporation of plasmid pJN105 (3), in PAO1.

Strains CF2162 [∆*dedA4* (P_ara_-empty)] and CF2164 [∆*dedA4* (P_ara_:: RBS_optimized_-*uppS*)] were obtained by electroporation of plasmids pJN105 (3) and pCF214 in CF1842 [∆*dedA4*], respectively.

Strains CF2168 [∆*ampD* (P_ara_-empty)] and CF2172 [∆*ampD* (P_ara_:: RBS_optimized_-*murA*)] were obtained by electroporation of plasmids pJN105 (3) and pCF166 in CF5 [∆*ampD*], respectively.

**References**

1. Rietsch A, Vallet-Gely I, Dove SL, Mekalanos JJ. 2005. ExsE, a secreted regulator of type III secretion genes in Pseudomonas aeruginosa. Proc Natl Acad Sci U S A 102:8006–8011.

2. Vvedenskaya IO, Sharp JS, Goldman SR, Kanabar PN, Livny J, Dove SL, Nickels BE. 2012. Growth phase-dependent control of transcription start site selection and gene expression by nanoRNAs. Genes Dev 26:1498–1507.

3. Newman JR, Fuqua C. 1999. Broad-host-range expression vectors that carry the L-arabinose-inducible Escherichia coli araBAD promoter and the araC regulator. Gene 227:197–203.

4. Choi K-H, Schweizer HP. 2006. mini-Tn7 insertion in bacteria with single attTn7 sites: example Pseudomonas aeruginosa. Nat Protoc 1:153–161.

5. Gyger J, Torrens G, Cava F, Bernhardt TG, Fumeaux C. 2024. A potential space-making role in cell wall biogenesis for SltB1and DacB revealed by a beta-lactamase induction phenotype in Pseudomonas aeruginosa. mBio.
